# A saccharide-based binder for efficient polysulfide regulations in Li-S batteries

**DOI:** 10.1038/s41467-021-25612-5

**Published:** 2021-09-10

**Authors:** Yingyi Huang, Mahdokht Shaibani, Tanesh D. Gamot, Mingchao Wang, Petar Jovanović, M. C. Dilusha Cooray, Meysam Sharifzadeh Mirshekarloo, Roger J. Mulder, Nikhil V. Medhekar, Matthew R. Hill, Mainak Majumder

**Affiliations:** 1 Nanoscale Science and Engineering Laboratory (NSEL), Department of Mechanical and Aerospace Engineering, Monash University, Clayton, VIC Australia; 2 Department of Materials Science and Engineering, Monash University, Clayton, VIC Australia; 3 CSIRO, Clayton, VIC Australia; 4 Department of Chemical Engineering, Monash University, Clayton, VIC Australia

**Keywords:** Energy, Batteries, Mechanical engineering, Batteries

## Abstract

The viability of lithium-sulfur batteries as an energy storage technology depends on unlocking long-term cycle stability. Most instability stems from the release and transport of polysulfides from the cathode, which causes mossy growth on the lithium anode, leading to continuous consumption of electrolyte. Therefore, development of a durable cathode with minimal polysulfide escape is critical. Here, we present a saccharide-based binder system that has a capacity for the regulation of polysulfides due to its reducing properties. Furthermore, the binder promotes the formation of viscoelastic filaments during casting which endows the sulfur cathode with a desirable web-like microstructure. Taken together this leads to 97% sulfur utilisation with a cycle life of 1000 cycles (9 months) and capacity retention (around 700 mAh g^−1^ after 1000 cycles). A pouch cell prototype with a specific energy of up to 206 Wh kg^−1^ is produced, demonstrating the promising potential for practical applications.

## Abstract (web summary)

The long-term cycling of Li-S batteries depends on the polysulfides shuttling regulation. Here, the authors present a saccharide-based binder system to control the polysulfides migration and improve the cycle life of a Li-S pouch cell.

## Introduction

Lithium-ion batteries (Li-ion) have changed the world. But as society moves away from fossil fuels at a massive scale, new battery chemistries with higher storage capacities and lower demands on critical minerals are going to be central^1^. At the same time, the viability of many emerging technologies, for example in aviation, require lighter-weight batteries. One such technology could be lithium-sulfur batteries (Li-S): which theoretically store as much as five times the energy of Li-ion and have a realizable specific energy of 400–600 Wh kg^−1^. They can be made from materials that are readily and sustainably available around the world. Until now, the realization of Li-S batteries has been challenging, mainly due to the instability of both electrodes, which results in a short cycle life of the battery. The power performance of the Li-S system is also inherently slow, particularly when the sulfur cathode is loaded to the required levels, mainly due to poor ion diffusion across the thickness of the cathode.

Extensive research over the past ten years has delivered marked improvements in the sulfur cathode, as well as a profound understanding of the failure mechanisms^2–6^. In 2010, Nazar et al. carried out pioneering research in the area of composite sulfur cathodes and addressed the challenge of the low-electrical conductivity of the sulfur cathode^7^. Later on, they reported on the introduction of polysulfide absorbents and mediators to the composition of the sulfur cathode and addressed the issue of ‘polysulfide shuttling’ to a large extent^8^. Quite recently, we have uncovered a pathway to overcome the structural instability of cathode, by introducing the expansion-tolerant architecture^9^. However, the problem of achieving high capacity simultaneously with extended cycle life has largely remained unsolved.

In stark contrast with Li-ion batteries, the solid electrolyte interphase (SEI) layer on the anode of the Li-S battery, while readily formed, also easily cracks due to polysulfide attack and the large swelling associated with lithium exchange. This leaves the freshly formed lithium surface in dynamic exchange with the polysulfide containing electrolyte^10^. The continuous reformation of the SEI is accompanied by the continuous consumption of the electrolyte. Eventually, the cell dries up and fails - presenting the biggest challenge to Li-S battery chemistry.

Li-S cycle life can be improved by using cathodes that can simultaneously accommodate the volume change and confine the polysulfides. To date several binder systems, such as natural gums^11,12^ and cellulose based binders^13–15^ have been explored to assist with the volume change. From these studies it can be inferred that cellulose-based binders serve well in fabricating mechanically robust cathodes. Furthermore, novel binder systems have been critically designed to add polysulfide absorbing functionality to the binder such as the electroactive nanocomposite binder composed of polypyrrole and polyurethane (PPyPU)^16^, and modified cyclodextrin (C-β-CD)^17^. The general conclusion from such studies for targeted retardation of polysulfide shuttling is that binders with polar/electronegative functional groups are beneficial to the sulfur cathode^16^. Unfortunately, these features have not yet delivered long term stable Li-S batteries because all prospective properties need to be combined within one system. Our cathode design concurrently provides expansion tolerance, strong polysulfide crossover limitation, and ion diffusion highways via nano-structuring—and it can be fabricated at scale from commonly sourced materials. These beneficial properties holistically mitigate the damage to the lithium metal anode, from which short circuits typically originate, ending the cycle life. The behaviour of these cathodes is emphasized by ex situ post-mortem analysis on the lithium anode of cycled cells. This demonstrated the lithium protection capabilities of our cathode that in turn delivered 1000 stable cycles over 9 months of continual operation.

Our current work is inspired by a 1988 geochemistry report^18^ that described how two saccharide-based substances, namely glucose and to a lesser extent cellulose, resist degradation in geological sediments by forming strong organo-sulfur bonds with polysulfides and hydrogen sulfide. Built on the strong binding ability of the high-modulus carboxymethyl cellulose (CMC) binder^19,20^, and the stronger ability of glucose for polysulfide regulation, we introduce a saccharide-based co-binder system that not only enables the fabrication of mechanically robust cathodes but also endows strong polysulfide confinement functionality. More importantly, our saccharide-based aqueous slurry promotes the formation of a web-like electrode microstructure. This can be rationalized by the viscoelastic filament thinning of the non-Newtonian binder systems and shaped by competing capillary forces and viscous drag. The result of the micro-architectural design leads to a segregated structure that rises to the challenge of stress evolution.

In this work, we exploit these synergistic effects to deliver a 97% sulfur utilisation with a cycle life of 1000 cycles and a high capacity retention (1106 mAh g^−1^ even after 500 cycles and around 700 mAh g^−1^ after 1000 cycles) while achieving >99% columbic efficiency (CE)—a clear demonstration of mitigating the damage on the lithium metal anode. To demonstrate the robustness of our binder system, we manufacture cathodes with a high loading of 10.5 mg cm^−2^, achieving an areal capacity of 12.56 mAh cm^−2^ with >98% CE. A pouch cell prototype with an initial capacity around 1200 mAh g^−1^ demonstrates a promising transition from laboratory to industry application.

## Results

### Effect of glucose on the polysulfide regulation

In stark contrast with the energy delivery mechanism in Li-ion, the liquid electrolyte and the sulfur cathode almost act like a couple to form the highly soluble polysulfide species, causing the well-known shuttle phenomenon. The weak nature of the physical interaction between the polar polysulfides and nonpolar fillers (carbon and binder materials)^21^ in a typical sulfur cathode cannot retard the shuttling effect over long-term cycling, a call for further functionalities to be incorporated into the cathode design. Here, we show that a saccharide-based binder system provides such functionalities.

**Fig. 1:**
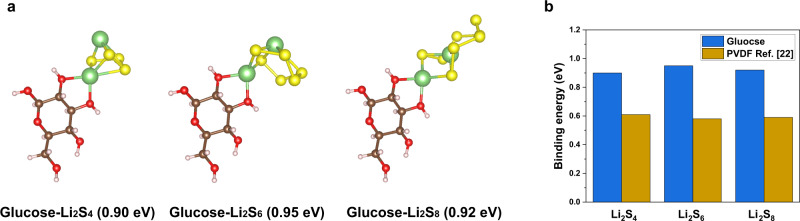
Simulation of LiPS adsorption. **a** Adsorption conformations and binding energies for Li_2_S_4_, Li_2_S_6_, and Li_2_S_8_ on glucose. **b** Binding energy comparison for glucose and the commonly used PVDF binder^22^ with various LiPS species, demonstrating the capacity of glucose for adsorbing polysulfides.

In order to gain an understanding of the interaction between saccharide-based binders and lithium polysulfides (LiPS), ab initio simulations performed in the framework of density functional theory were carried out to investigate the adsorption of LiPS species on glucose. As shown in Fig. 1a, a strong interaction with LiPS and hydroxyl groups within the binders was observed. In all these coordination complexes (glucose and LiPS), the most stable configuration corresponds to lithium binding directly to oxygen atoms and forming lithium–oxygen bonds, with binding energies of 0.90 eV for glucose-Li_2_S_4_, 0.95 eV for glucose-Li_2_S_6_ and 0.92 eV for glucose-Li_2_S_8_. Other two possible binding sites in glucose were considered and summarized in Supplementary Fig. 1, have binding energies from 0.72 to 0.94 eV. Compared to the binding energy between the same LiPS species and commercially available binder for Li-S battery—polyvinylidene fluoride (PVDF), shown in Fig. 1b, the total binding energies range from 0.58 to 0.61 eV^22^, much lower than those of glucose. The binding energies and corresponding adsorption conformations between CMC and LiPS also calculated and demonstrated in Supplementary Fig. 2.

**Fig. 2:**
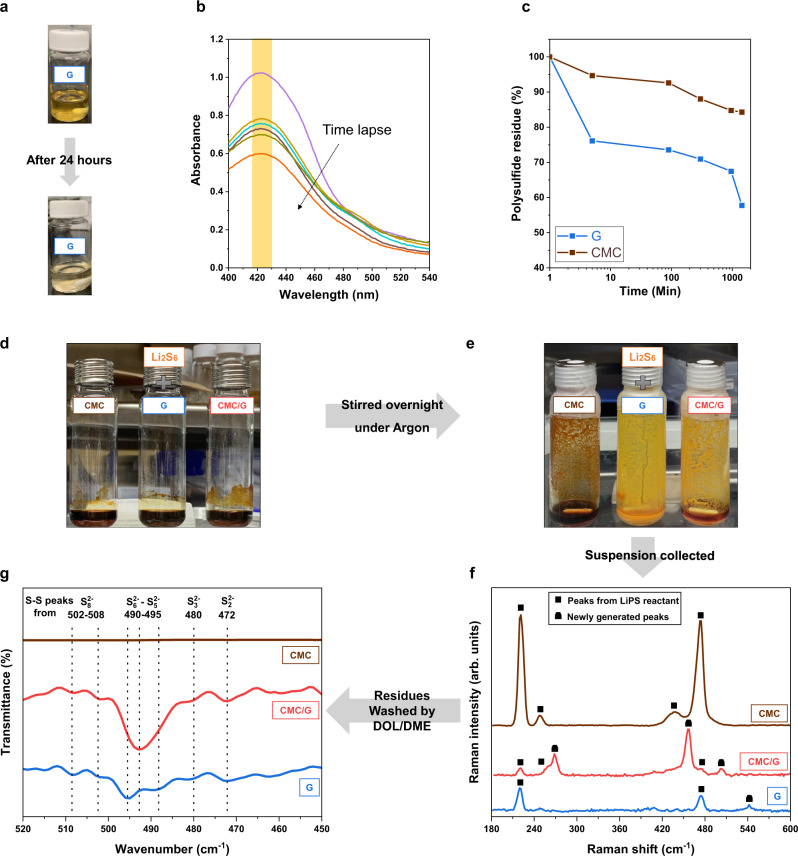
Polysulfide adsorption study. Adsorption tests via UV–Vis. **a** Evolution of polysulfide with glucose in DOL/DME electrolyte solution; **b** UV–Vis spectrum of Li_2_S_6_ with glucose in DOL/DME electrolyte solution after a specific time; **c** comparison of LiPS adsorption between CMC and glucose. **d**, **e** Illustrating the evolution of polysulfide in the presence of high concentrate lithium polysulfide; **f** Raman spectra of suspensions and **g** FTIR spectra of washed solid residues.

To further examine the ability of saccharide-based materials for LiPS regulation, several comparative tests were carried out with glucose and CMC in presence of LiPS solution. Ultraviolet–visible spectroscopy (UV–Vis) detected the residue concentration of polysulfide after different incubation times. The peak of LiPS was detected at a wavelength around 420 nm in agreement with literature^23^. The results showed that 42% of polysulfide eventually adsorbed in the presence of glucose (Fig. 2a and b), compared to only 16% in CMC with the same weight percentage of absorbent (Supplementary Fig. 3), demonstrating almost three times more capacity for LiPS adsorption (Fig. 2c). In addition, two glass transparent Li-S cells were assembled, shown in Supplementary Fig. 4, the difference of electrolyte colour in two cells after cycling were apparent, which demonstrate the ability of the CMC/G cathode to retain LiPS.

Further analysis was carried out on the suspension obtained from the reactions between a highly concentrated LiPS solution and glucose (G), a mixture of 67 wt. % of CMC and 33 wt. % of G (CMC/G), and CMC composition to uncover the possible role of glucose in the polysulfide reducing reactions. Figure 2d and e show the vials after overnight incubation. These suspension samples were drop cast on glass slides, dried under Argon inside the glove box and collected for Raman spectroscopy analysis to study polysulfide speciation. Four peaks can be found in the LiPS + CMC sample (Fig. 2f, brown line). The peaks at 221 cm^−1^ and 472 cm^−1^, can be attributed to unreacted crystalline sulfur (S_8, solid_)^24^ from LiPS synthesis and peaks at 247 cm^−1^ and 435 cm^−1^ are related to higher order LiPS (Li_2_S_6_ and Li_2_S_8_)^25–27^ in agreement with the literature. A control sample of highly concentrated LiPS was examined under Raman spectroscopy (Supplementary Fig. 5a), which showed peaks similar to the LiPS + CMC sample. This comparison demonstrates that the CMC, as expected, cannot carry forward the reducing reactions of higher order LiPS to the lower orders ones. In contrast, the spectra of glucose containing samples show reduced intensity of these four peaks, indicating lower content of elemental sulfur or high order LiPS. In stark contrast, new peaks in the LiPS + CMC/G sample spectrum can be observed at 268 cm^−1^, 503 cm^−1^ and 457 cm^−1,^ being associated with S_4_^2−^, S_4_^−^ and S_6_^2−^ respectively^18–26^. For the LiPS + G sample, the peak at 542 cm^−1^ can be attributed to S_3_^∙−^ ^24,28^. The presence of newly generated lower order LiPS in the glucose containing samples (LiPS + G and LiPS + CMC/G residues) conveys the strong ability of glucose to encourage the conversion of higher order LiPS to lower order, more reduced LiPS, given that glucose is a well-known reducing agent^29^. These functions are often linked with high capacity and enhanced capacity retention^30^.

Further analysis was undertaken using Fourier transform infrared (FTIR) spectroscopy, which provides information on different LiPS species by monitoring the vibrational modes of S–S bonds^31^. The suspensions were vacuum filtered, and the solid residues were collected. These solid residues were substantially washed with 1, 3-dioxolane (DOL) and 1, 2-dimethoxyethane (DME) (1:1, v/v) to remove higher order soluble LiPS or weakly bonded LiPS to CMC or glucose. After drying under vacuum, FTIR spectroscopy was performed on dry solid residues. The peaks of LiPS species^32^ can be observed primarily between 450 and 520 cm^−1^. As shown in Fig. 2g, the spectrum is conspicuous by the absence of S–S peaks in the LiPS + CMC solid residue. This could be a result of weak interaction between LiPS and CMC, which causes the LiPS to wash away while collecting the solid residue. In contrast, in the samples, which contained glucose (LiPS + G and LiPS + CMC/G solid residue samples), the S–S vibrational modes can be observed for a range of LiPS species. The peaks at 502 cm^−1^ and 508 cm^−1^ can be attributed to S_8_^2−^ species^31^, while those at 490–495 cm^−1^ are attributed to S_6_^2−^ and S_5_^2−^ species^31^ and at 480 and 472 cm^−1^ are due to the presence of S_3_^2−^ and S_2_^2−^ in agreement with the previous reports^31,33^. As expected, control FTIR analysis on CMC powder, glucose powder and CMC/G composite film (Supplementary Fig. 5b), didn’t show any peaks in the range of 450–520 cm^−1^. The Full FTIR spectra for residue samples are shown in Supplementary Fig. 5c. This demonstrates that the peaks observed in glucose containing samples are due to the interactions between glucose and LiPS species. As a control, the FTIR spectrum of higher order LiPS solution is shown in Supplementary Fig. 5d.

Utilizing three independent techniques, viz. Raman, FTIR, and UV–Vis spectroscopies, we showed that the monosaccharide (glucose) has a distinct role in enhancing the polysulfide adsorption and interaction capacity compared to the polysaccharide (CMC) alone. We attribute this to the increased and more accessible active reaction sites compared to its polymeric counterpart, since compared to CMC, glucose is a monomer and has more free binding sides with higher degrees of freedom, which means it is more chemically active than CMC. In addition, the enhanced conversion of higher order to lower order LiPS, as evident by Raman and FTIR, is expected to improve the battery chemistry by slowing down the shuttling effect of polysulfides.

**Fig. 3:**
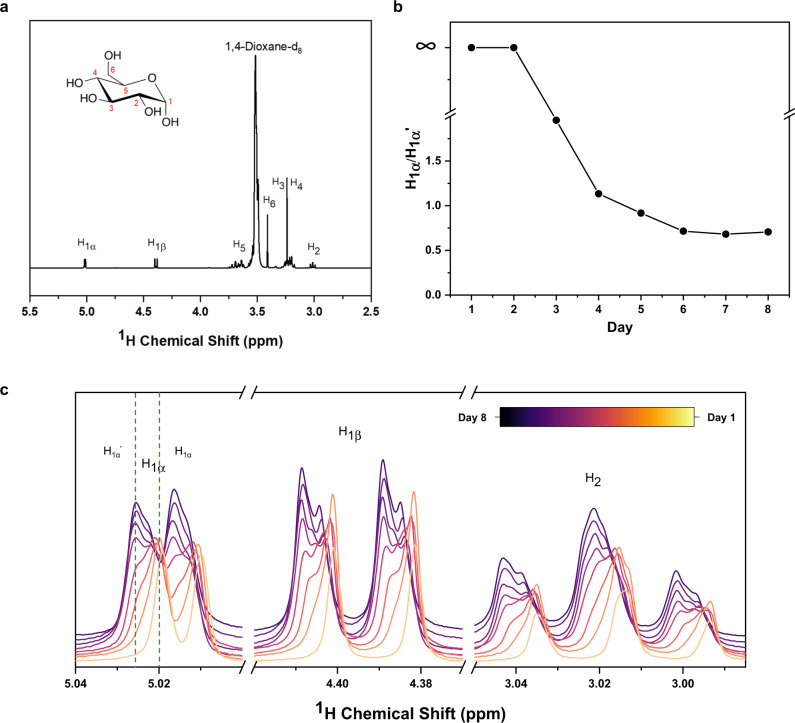
^1^H NMR analysis probing the glucose–Li_2_S_6_ interactions within a simulated battery environment. **a** Full ^1^H NMR spectrum for the glucose/ Li_2_S_6_ composites. **b** The proportional evolution between H_1α_ and H_1α_ˈ over 8 days. **c** ^1^H NMR spectra over 8 days.

To further study the interaction between polysulfide and glucose, in situ Nuclear magnetic resonance (NMR) experiments were conducted on CMC or glucose powders in presence of lithium polysulfide in dioxane-d_8_ solvent for 8 days to simulate battery environment. The full ^1^H NMR spectrum for the glucose and lithium polysulfide composites is shown in Fig. 3a. It confirms that the signals received primarily originate from the glucose binder present. ^1^H NMR spectra were collected over 8 days. A downfield shift is observed across all signals, indicating increased interaction with electron withdrawing groups as the battery reactions proceeded, and is strongest for peaks H_1_ and H_2_, indicating the site of this interaction. The modelling highlighted in Fig. 1 and binding side 2 in Supplementary Fig. 1 indicated H_1_ and H_2_ as the preferred sites for intercalation of lithium polysulfides, in strong agreement with the NMR results. This shift was quantified by an in-depth examination of the H_1α_ signal, as shown in Fig. 3b and c. Fitting of the integrated peaks (Supplementary Fig. 6) revealed a significant growth in the lithium polysulfide coordinated analogue, denoted as H_1α_ˈ that stabilised after 6 days. These results confirm that glucose plays an active role in the immobilisation of polysulfides and contributes to the increased lifetime of the battery.

### Preparation of sulfur electrodes with saccharide-based binders

Adhering to the conventional cathode processing methods and commonly sourced materials, we manufactured our cathodes by mixing carbon black, crystalline sulfur and different proportions of CMC and glucose as the binder system in deionized (DI) water. To bring the innovative binder formulation into context, we notice that CMC is a commonly used binder in the fabrication of battery electrodes. Our recent work demonstrates that controlling the dispersion of CMC enables the formation of mechanically strong bridging bonds between the colloidal sulfur particles and conductive carbon to produce cathodes with a unique expansion-tolerant (ET) architecture^9^. This design, while very effective in terms of accommodating the cycling stress, has limited contribution in regulating the shuttle of polysulfides. As such, the damage on the lithium as a result of the constant attack of the polysulfide is inevitable and the batteries cannot perform to their high capacity over long term cycling. This calls for significantly more functionality to be introduced to the cathode from the binder, other than only assisting with the stress management.

We fabricated four types of sulfur cathodes with identical fractions of the components [70 wt. % sulfur, 20 wt. % carbon, and 10 wt. % binder system] yet using different fractions of glucose or CMC in the binder system, as explained in Table 1.

**Table 1:** Description of the cathodes with different binder systems.

Code	Components and proportion				Zero-shear viscosity of binder (Pa·s)
	CMC	Glucose (G)	Sulfur (S)	Carbon(C)	
Pure CMC (common practice)	10%	0	70%	20%	736
0.67CMC + 0.33G (our work)	6.67%	3.33%			8.23
0.5CMC + 0.5G (control experiment)	5%	5%			0.73
Pure G (control experiment)	0	10%			0.06

**Fig. 4:**
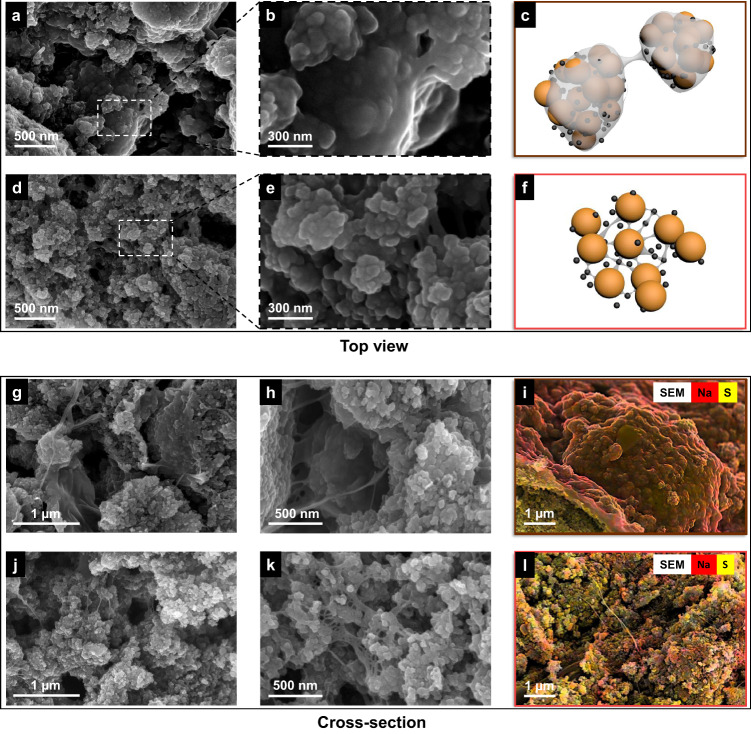
Microstructural study, elemental mapping, and schematic illustration of the sulfur electrode with the different binder systems. Top-view SEM images and schematic illustration of the architecture in sulfur cathodes with different binders. **a**–**c** pure CMC as the binder, demonstrating a cohesive network of agglomerated particles being trapped in the network of the binder; **d**–**f** CMC/G as the binder, illustrating a segregated structure that separated particles linked by web-like binders. Cross-sectional SEM images and elemental mapping of cathodes with different binders. **g**–**i** pure CMC as the binder, demonstrating a continuous binder film covering on the surface of particles; **j**–**l** CMC/G as the binder, illustrating the existence of bridging bonds across the electrode and excellent sulfur exposure.

We conducted detailed scanning electron microscopy (SEM) studies at a wide range of magnifications to investigate the morphology of these cathodes. Figure 4a–c illustrates a typical CMC-based cathode architecture. As evident in Fig. 4a and b, and as shown in the schematic figure (Fig. 4c), the cling wrap-like binder film covers active and conductive particles over a large area. For a desired architecture of the electrode, all active particles should be uniformly distributed within the conductive network of the electrode to enable homogeneous utilization of the active material. Further, to allow for facilitated electrolyte penetration, uniformly distributed low-resistance internal pathways are also critical^34^. In stark contrast with the CMC-based cathode, the cathode with CMC/G binder system displays an advantageously more segregated structure (Fig. 4d–f for the top view). Sulfur and carbon are exposed to a large extent owing to dispersed particle-level link instead of an agglomerated network. This web-like structure endows the sulfur cathode with the maximum exposure of the active materials, enhanced electrolyte accessibility and low resistance as well as short internal pathways for lithium-ion transfer.

The cross-sectional SEM images and energy dispersive X-ray (EDX) mapping (Fig. 4g–i) of the same electrode also provides evidence of the continuous coverage of CMC binder over particles, which blocks off vital transport pathways. In contrast, the cross-sectional SEM images and EDX mapping shown in Fig. 4j to l provide evidence for the formation of web-like structure in the CMC/G cathode.

### Mechanistic insight and viscoelastic analysis of the saccharide-based binder system

**Fig. 5:**
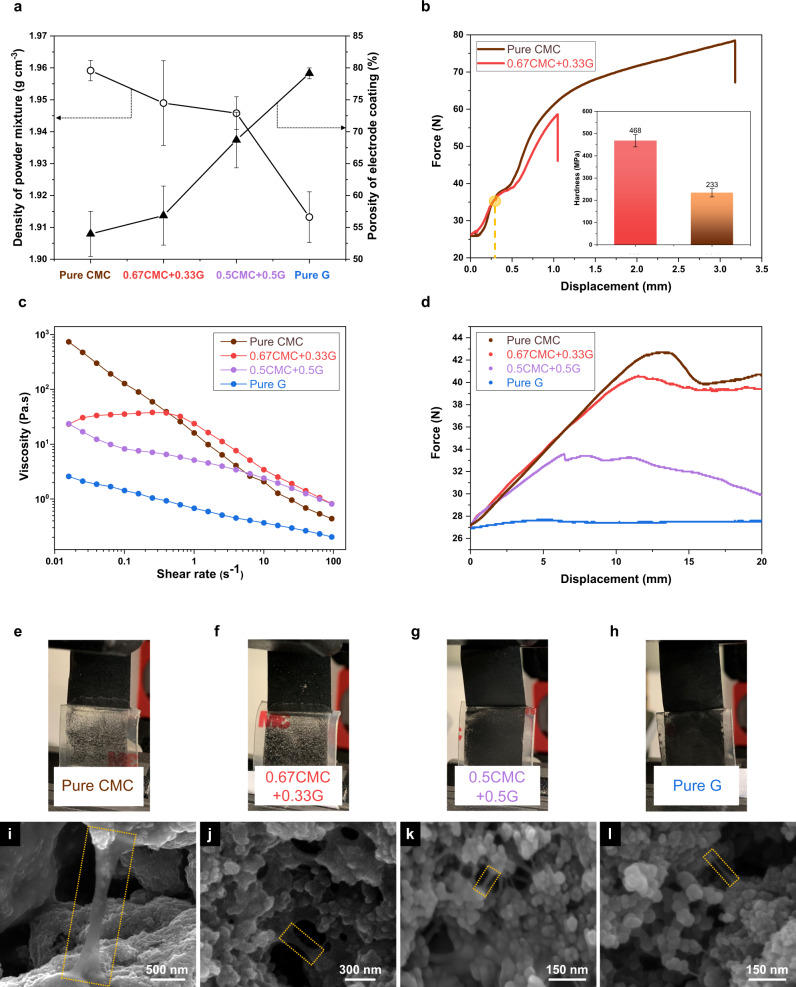
Physical, rheological and filament thinning properties. **a** Density of powder mixture including sulfur, carbon and binder, and porosity of the final electrode among four different cases. The error bars (standard deviation) of density were calculated based on 10 sets of data and the error bars (standard deviation) of porosity were calculated based on 5 sets of data. **b** Tensile test and indentation test of cathodes with CMC/G and pure CMC as binder. **c** Steady-state shear flow behaviour. Peeling test **d** Force versus displacement plots of the peeling test among four samples; **e**, **f** Photos of the peeling test setup and **i**–**l** microstructures of binder for corresponding samples (**e** and **i** cathodes with pure CMC as binder; **f** and **j** cathodes with 0.67CMC + 0.33 G as binder; **g** and **k** cathodes with 0.5CMC + 0.5G as binder; **h**, **l** cathodes with pure glucose as binder).

To obtain a mechanistic insight into the cathode microstructure, we quantified the critical physical properties of both the slurry and the coating. The apparent density of four different cathode mixtures was measured by gas pycnometer. The following equation^35^ was employed for estimating the porosity of the electrode.

1 $$\rho =\frac{V({{{{{\rm{cathode}}}}}})-{V}_{{{{{{\rm{dense}}}}}}}({{{{{\rm{cathode}}}}}})}{V({{{{{\rm{cathode}}}}}})}$$

where *V*(cathode) is the geometric volume of the electrode calculated using the thickness of the cathode as measured by cross-section SEM and depicted in Supplementary Fig. 7. *V*_dense_ (cathode) is the dense volume of the cathode, calculated by the measured mass of the coating and dividing it by the apparent density of all the cathode components as determined by gas pycnometer. The results displayed in Fig. 5a, illustrate that the porosity of cathode increased with increasing content of glucose. The mechanical test result (Fig. 5b) showed that the hardness of CMC film was enhanced by employing glucose, the overall rupture point decreased, but importantly, the force required for small displacements (less than 250 µm) was increased by adding glucose. The combination of the elevated hardness and increased force requirement for small scale deformation of CMC/G composite, suggest a binder system for managing the ~80% volume change of the sulfur electrodes.

To the best of our knowledge, a clear evaluation standard of rheological properties for electrode slurries is not available in the literature. Based on the viscoelastic filament study of the binder and the corresponding performance of batteries (Supplementary Fig. 8) in this research, we propose using the Ohnesorge (Oh) number^36^ as a selection guide for binders to generate the web-like and well-segregated architecture. In below, we will discuss that the binder system with Oh number >1 and within double-digit is optimal for web-like network formation which helps to develop a segregated structure within the electrode.

The physical properties of cathode slurries were evaluated using the rheological tests. The steady-state shear rheology, depicted in Fig. 5c, shows that while the slurry with CMC and glucose as co-binder has lower apparent viscosity, it has a lower degree of shear thinning characteristics as evident from the fit of the power law model to the steady-state shear flow measurements (Supplementary Fig. 9). The sudden change in the value of flow behaviour index (n) with the addition of glucose (pure CMC vs. 0.67CMC + 0.33G) can be accounted for the reduction in the flow behaviour, from increased molecular interactions of the small glucose molecules with the polymeric chains of CMC. This means that under high shear stress, such slurries are less likely to deform^37^.

Apart from flow properties, the dynamic behaviour of slurries plays a vital role in stability and mechanical properties of the binder and the binder-solid particle interaction. The principles of viscoelastic filament thinning, and breakup come into effect by the action of viscous drag and capillary forces. When the binder liquid is stretched during the process of mixing with solid particles, a string or filament of liquid forms between them. On solidification, the filament is consolidated if the filament can retain the stability before drying, and this phenomenon determines how the bridge-like network is formed. To rationalize the formation of the filamentous structures in the cathode, we used a dimensionless number, Ohnesorge number.

2 $${{{{{\rm{Oh}}}}}}={{{{{{\rm{\eta }}}}}}}_{0}/\sqrt{{{{{{\rm{\rho }}}}}}{{{{{\rm{R}}}}}}{{{{{\rm{\gamma }}}}}}}$$

which represents the relative importance of viscous with inertial and capillary forces^38^. Here *η*_0_ is zero-shear viscosity, *ρ* is density, *γ* is the surface tension of the liquid and R is the radius of the filament^39^. The magnitude of Oh can designate three different morphologies of filamentous structures. For viscous filaments with Newtonian behaviour and Oh > 1, the formation of beads-on-a-string (BOAS) morphology is predicted. In the case of non-Newtonian filaments, elasticity disrupts the formation of the beads giving axially uniform shape. For low-viscosity filaments with Oh < 1 inertially and capillary dominated slender thread type morphology forms which can become non-uniform with time^36^.

Using SEM micrographs, the average and mean radius of the filaments were deduced by plotting log-normal and gaussian distribution curves (Supplementary Fig. 10 and Supplementary Table 1). The mean radius x_c_ and standard deviation w stand high at 0.04 microns and 0.30, respectively for pure CMC binders. With the addition of glucose, the mean, as well as standard deviation decreases indicating filament thinning behaviour.

The estimated Ohnesorge number for the four binder systems (without sulfur and carbon) are tabulated in Table 2 (Zero-shear viscosity of four binders shows in Supplementary Fig. 11a, surface tension of four binders shows in Supplementary Fig. 11b and density of four binders shows in Supplementary Table 2). It was found that for pure CMC and 0.67CMC + 0.33G, Oh > 1 while for 0.5CMC + 0.5G and pure G, Oh < 1. Pure CMC with Oh = 547.03 >> 1 shows viscosity dominated filament formation. Our amplitude sweep measurements in Supplementary Fig. 11c support the viscoelastic nature of pure CMC and 0.67CMC + 0.33G binder systems. The extent of linear viscoelastic (LVE) regime suggests that pure CMC has a solid-like (elasticity-dominant) behaviour. With large viscous drag, viscosity-dependent elongation ensures the filaments are axially uniform with a large mean radius and high elasticity maintains the large radius after the elongation has ceased. In general, the addition of glucose to the CMC system decreases not only the viscosity (Fig. 5c), but also the elasticity. For example, the difference between storage modulus *G*′ and loss modulus *G*′′ (Supplementary Fig. 11c) for 0.67CMC + 0.33G is smaller than pure CMC, indicating a decrease in elasticity-dominant behaviour. The decrease in elasticity can be further confirmed by frequency dependence of *G*′ in our frequency sweep measurements (Supplementary Fig. 11d). The applied forces here are of capillary nature, which dominates over viscous drag (Oh ~ 8) and elasticity leading to thinner and axially uniform filaments.

**Table 2:** Ohnesorge number calculation.

Binder filaments	*η*_0_ (Pa.s)	*ρ* (kg m^−3^)	R (µm)	*γ* (mN m^−1^)	Oh
Pure CMC	736.00	1029.97	0.043	40.64	547.03
0.67CMC + 0.33G	8.23	1026.10	0.017	54.64	8.46
0.5CMC + 0.5G	0.73	1024.67	0.011	59.85	0.89
Pure G	0.06	1024.60	0.007	58.89	0.10

For 0.5CMC + 0.5G and pure G, Ohnesorge number Oh < 1, indicates the domination of inertiocapillary forces as demonstrated by low-viscosity fluid filaments leading to thinner filaments (mean radius *x*_*c*_ = 0.011 microns and 0.007 microns for 0.5CMC + 0.5G and pure G respectively)^40^. Additionally, these slurries show liquid-like behaviour with *G*′′ higher than *G*′ in the amplitude sweep measurement (Supplementary Fig. 11c) and supported by frequency sweep measurements (Supplementary Fig. 11d) indicating loss of elastic nature likely from broken down molecular network rendering a more liquid-like behaviour^41^. Amplitude sweep measurements of CMC and G binder itself with different solid content in water show in Supplementary Fig. 11e. The addition of glucose to the polysaccharide binder decreases the viscosity and elasticity and promotes the web-structure of thin filaments between the particles by maintaining a balance between viscous and capillary forces which otherwise would form an agglomerated network of particles.

To evaluate the adhesion and cohesion strength of electrodes, peeling test among four samples is performed^42^. Mounting tape is pressed intimately on the surface of the electrodes and removed with a steady motion. As shown in Fig. 5d, the force required to peel off the tape on electrode is decreasing with the increasing amount of glucose added to the binder system. In the meantime, more particles peel off from the electrode (Fig. 5e–h), which are associated with the reduced radius and weakened binder filaments (Fig. 5i–l).

### Li-S coin and pouch cells testing and ex situ post-mortem investigations

**Fig. 6:**
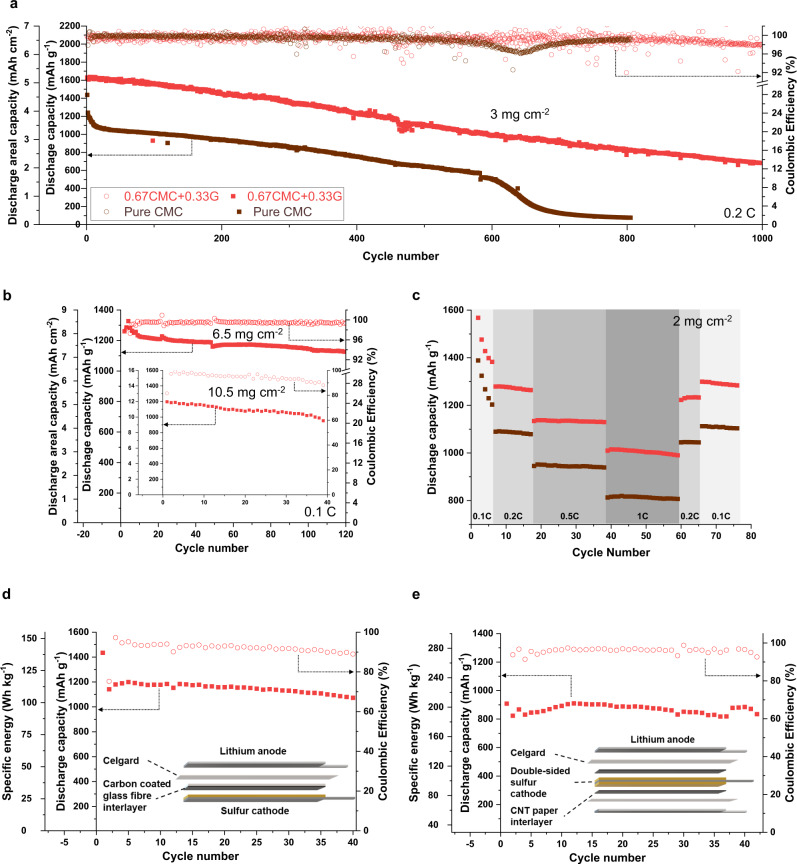
Cycling performance comparison between CMC cathode and CMC/G cathode. Coin cells configured with **a** 3 mg cm^−2^ sulfur loading cathode, **b** 6.5 mg cm^−2^ sulfur loading cathode and 10.5 mg cm^−2^ sulfur loading cathode shown in the insert plot. **c** Rate capability data among two compared coin cells (2 mg cm^−2^ sulfur loading). Red lines indicate the performance of CMC/G cathode, and the brown lines indicate the performance of CMC cathode. Configuration of the pouch cell with **d** single-sided cathodes **e** double-sided cathodes with lean electrolyte condition (E/S = 6 μL mg^−1^), optimised for specific energy. 1 C-rate is equivalent to 1 h charge or discharge a battery. To convert C-rate to current density, current density (mA cm^−2^) = sulfur loading (g cm^−2^) × Li-S theoretical capacity (1675 mAh g^−1^) × C-rate.

To verify the impact of glucose on cycling performance, we prepared cathodes with different compositions (Table 1) and sulfur loadings. For a cathode with CMC/G binder system (sample 0.67CMC + 0.33G) and sulfur loading of 3 mg cm^−2^, we achieve a 97% sulfur utilisation, delivering an initial capacity of 1629 mAh g^−1^ with above 99% CE and an areal capacity of 5.1 mAh cm^−2^ at 0.2C (Fig. 6a). Importantly, this cathode demonstrates long cycle life while maintaining high reversible capacity, 1106 mAh g^−1^ after 500 cycles (68% capacity retention) and around 700 mAh g^−1^ after 1000 cycles (42% capacity retention), over 9 months of continual operation. In contrast, the cathode with pure CMC binder shows 23% lower initial capacity and whilst delivering stable performance over the first few hundreds of cycles, demonstrates a capacity decay after around 580 cycles. The capacity retention ability of both cathodes over the first 500 cycles demonstrates the capability of the CMC as a binder that enables the fabrication of robust cathodes. However, it is only in the presence of glucose with its polysulfide regulation capability and web-like network formation that achieving 1000 high-capacity cycles is possible.

In coin cell level, the CMC/G binder system can be used to successfully fabricate high sulfur loading electrodes (6.5 mg cm^−2^) which shows high specific capacity above 1200 mAh g^−1^ with 120 stable cycle life. For a sulfur cathode loading of 10.5 mg cm^−2^, the coin cell delivers 12.56 mAh cm^−2^ areal capacity and high CE, >98%, depicted in Fig. 6b. The plots with more detail variation of CE upon cycling are shown in Supplementary Fig. 12. Quite importantly, the CMC/G cathode delivers better rate capability performance compared to that of CMC cathode, around 1000 mAh g^−1^ at 1C cycle rate (Fig. 6c). In addition, as shown in Supplementary Fig. 13a, with the increase in sulfur loading, the specific capacity demonstrates a retention. For instance, at 5 mg cm^−2^, the cathode delivers specific capacity of 1256 mAh g^−1^ and, at 10.5 mg cm^−2^, it still delivers a specific capacity as high as 1189 mAh g^−1^. In Supplementary Fig. 13b and Supplementary Table 3, we have drawn a performance comparison in the literature of high cycle life Li-S cells, >500 stable cycles^2,43–55^. As can be seen, our cathodes demonstrate performance in the combined metrics of areal capacity and cycle life.

In pouch cell level, the prototype with an initial capacity 1200 mAh g^−1^ shown in Fig. 6d demonstrates the scalability of the cathode production. As shown in Fig. 6e, the prototype with optimized configuration and leaner electrolyte condition achieves specific energy of up to 206 Wh kg^−1^ while demonstrating a great stability, indicates the potential for a successful translation from laboratory to industrial production. The parameters and calculated proportion of components in these two pouches are shown in Supplementary Fig. 14.

**Fig. 7:**
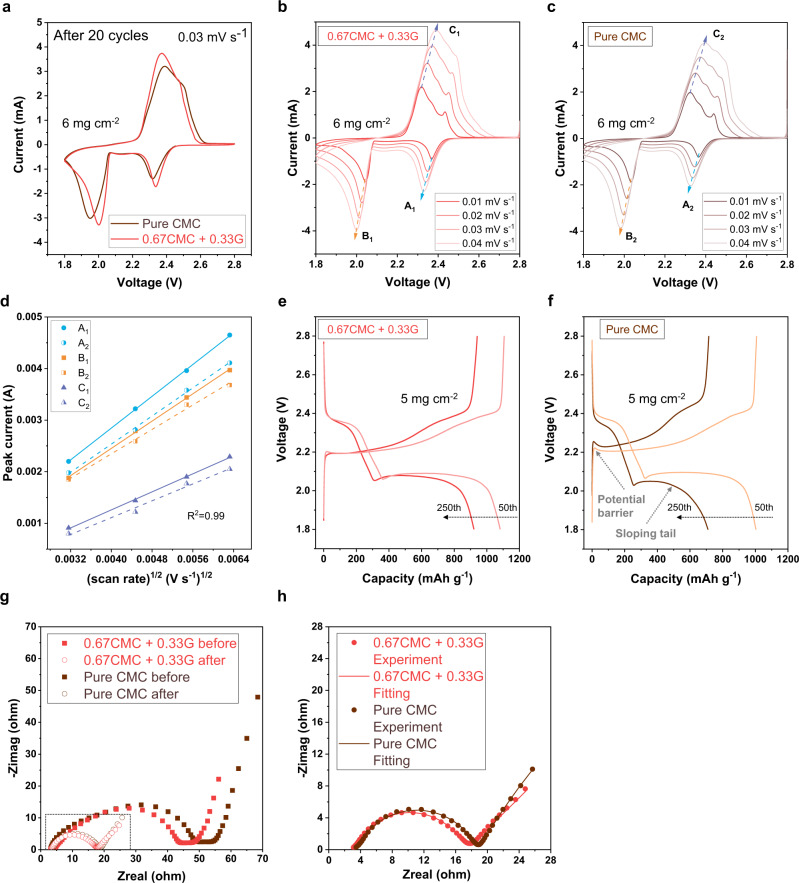
Electrochemical characterisation on sulfur cathodes with two different binder systems. Cyclic voltammogram profiles. **a** CV profiles comparison; CV profiles at different scan rates of lithium-sulfur cells with **b** CMC/G cathode and **c** CMC cathode; **d** the linear fits (*R*^2^ = 0.99) of the CV peak currents for the lithium-sulfur cells with CMC/G cathode (A_1_, B_1_, C_1_) and with CMC cathode (A_2_, B_2_, C_2_). Charge/discharge profiles corresponding of lithium-sulfur cells with **e** CMC/G cathode and **f** CMC cathode. Electrochemical impedance spectroscopy **g** Nyquist plots of the lithium-sulfur cells with CMC/G and CMC cathodes before and after 80 cycles; **h** Nyquist plot and equivalent circuit fitting of cells after cycling.

Electrochemical behaviour of coin cells configured with CMC/G and CMC cathodes is further studied by analysing their cyclic voltammogram (CV), charge/discharge profiles and electrochemical impedance spectroscopy (EIS) spectra. The EIS and CV tests of glucose cathode are shown in Supplementary Fig. 15. The CV profiles of both cells after 20 cycles exhibit two major reduction peaks around 2.3 V and 2.0 V, as depicted in Fig. 7a. Theoretically, the peak at higher cathodic voltage is related to the reduction of sulfur to high order LiPS (Li_2_S_n_, 4 ≤ n < 8), and the peak at the lower voltage is associated with the conversion of higher order LiPS to lower order LiPS (Li_2_S_2_ and Li_2_S). The reactions are reversed in the anodic scan. CMC/G cathode displays higher magnitude of cathodic and anodic peaks, demonstrating enhanced lithiation/delithiation kinetics^56^. Moreover, the CMC/G cathode exhibits the reduction peaks at a relatively higher voltage range compared to the CMC cathode, suggesting lower resistance of the electrochemical reaction^57^. As shown in Fig. 7b and c, below, identical cells with CMC/G cathode and CMC cathode (6 mg cm^−2^ sulfur loading) were made for lithium-ion diffusion coefficient test. A series of CVs with different scan rates were used for calculation according to the Randles-Sevick equation (Fig. 7d)^46,58^. The values of lithium-ion diffusion coefficient were evaluated to be 1.47 × 10^−7^ cm^2^ s^−1^ to 4.56 × 10^−7^ cm^2^ s^−1^ for lithium-sulfur batteries with CMC/G cathode (Fig. 7b), and 1.28 × 10^−7^ cm^2^ s^−1^ to 3.57 × 10^−7^ cm^2^ s^−1^ for CMC cathode (Fig. 7c). The elevated lithium-ion diffusion coefficient for CMC/G cathode confirms the enhanced lithiation/delithiation kinetics of sulfur cathode using CMC/G binder system.

The 50^th^ and 250^th^ charge/discharge profiles of the two cells with 5 mg cm^−2^ sulfur loading are plotted in Fig. 7e and f. In the charge profiles of the cell with CMC cathode, distinct potential barrier occurred at the beginning of the charging process, demonstrating the presence of insulating Li_2_S_2_ and Li_2_S deposited on the electrode surface and greater polarization^56^. Furthermore, the 250th discharge plot of the cell with CMC cathode shows a sloping tail which is associated with the solid to solid reduction of Li_2_S_2_ to Li_2_S^56^. When the Li_2_S_2_ formation dominates the final step of the reduction to Li_2_S, rather than Li_2_S_n_ (4 ≤ n < 8), the dynamics of the electrochemical conversion are more sluggish due to the retarded diffusion kinetics in solid-state. This phenomenon is relieved to a large extent in the cell with CMC/G cathode. The possible explanation is that the web-like structure and increased porosity of the CMC/G electrode provides mass transfer highways for electrolytic species.

EIS measurements are carried out to verify the alternating current (AC) impedance of the two cells before and after 80 cycles (Fig. 7g and Fig. 7h) by fitting the Nyquist plots with the equivalent circuit (Supplementary Fig. 16)^59,60^. The equivalent circuit features electrolyte resistance, two RC (resistance and constant phase element) parallel elements in series representative of the resistance of the solid electrolyte interphase and charge-transfer, and the Warburg diffusion impedance corresponding to the diffusion of Li-ion on the interfaces between electrolyte and electrodes^59^. It shows that the CMC/G cathode yields lower charge-transfer resistance which is consistent with the reduced internal resistance and enhanced ionic transfer in the web-like network of the cathode architecture.

**Fig. 8:**
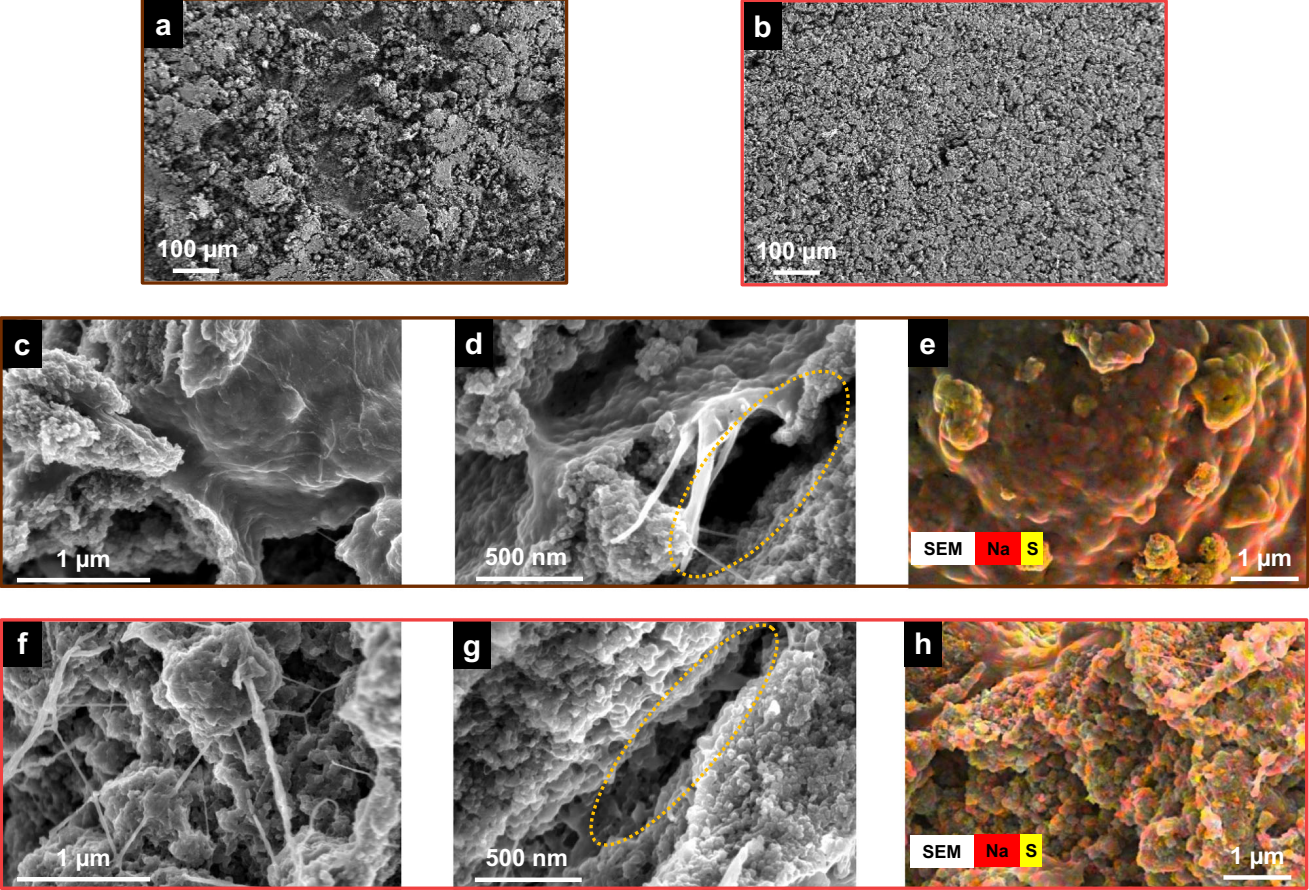
Ex situ post-mortem of lithium metal anode and sulfur cathode after an intense cycling regime. Top-view SEM images of lithium metal coupling with **a** CMC cathode and **b** CMC/G cathode. Cross-sectional observation and elemental mapping of **c**–**e** CMC cathode and **f**–**h** CMC/G cathode at full charge state.

Finally, further evidence of the LiPS regulation ability of glucose is observed by the ex situ post-mortem SEM of the cycled lithium anodes and sulfur cathode. After an intense 100 cycles of rate capability test from 0.1C to 1C, cells were disassembled and lithium metal anodes and sulfur cathode were washed by DOL/DME and collected for SEM imaging. As shown in Fig. 8a, the surface of the lithium metal from the cell with CMC cathode is severely corroded. On the other hand, for the lithium anode coupled with CMC/G cathode (Fig. 8b), the corrosion is considerably reduced, and more uniform surface topography is noticeable. The SEM images and EDS mappings of the full charge/delithiation state of both CMC and CMC/G cathode are presented in Fig. 8c–h. The lower magnification images shown in Fig. 8c and f show no obvious microstructural changes for both the cling wrap-like binder film in CMC cathode and the web-like binder structure in CMC/G cathode. Nevertheless, more structural details are provided by higher magnification images. CMC cathode (Fig. 8d) demonstrates the evolution of large crack as cause of delamination between binder film covered surface layer and the layer underneath, which is a clear structural disintegration after exposure to intense cycling stress. In contrast, the CMC/G cathode (Fig. 8g) demonstrates preserved binders between particles. Benefited from the segregated microstructure for accommodating the volume changes during cycling, CMC/G cathode develops no major cracks after cycling. The EDS mappings (Fig. 8e and h) confirm general structural integrity of both binders after cycling.

## Discussion

To date, the main function expected from different binders used in the fabrication of sulfur cathodes has been to glue the carbon and sulfur together and make crack-free structures. We have shown that a simple saccharide-based binder system can impart two critical functionalities to a non-sophisticated sulfur cathode, polysulfide regulation and controlled porosity, leading to a stable cycle life.

The significant contributions of glucose can be summarised into two aspects. Firstly, glucose, being a strong reducing agent, enables the conversion of higher order LiPS to lower order LiPS, while also enhancing the LiPS retention capacity – these properties improve the battery chemistry by slowing polysulfide shuttling. Secondly, glucose has a strong role as a viscosity modifier of the binder liquid, with order-of-magnitude changes recorded. This allows the viscoelastic filaments to be desirably shaped during a typical electrode formation process. By fine tuning the microstructure for improved electrolyte access and ion transport properties, alongside increased polysulfide retention capacities, our CMC/G cathodes show dramatically enhanced capacities and cycle life. We offer generic insights into how the mechanically strong segregated structures can be guided by the choice of viscoelastic features and criterion of the fluid properties. The combination of the optimal chemical and mechanical aspects of the binder chemistry leads to significantly enhanced Li-S batteries with high specific capacity and long cycle life of initial 1629 mAh g^−1^ and 1000 cycles, respectively. The pouch cell prototype indicates that our approach of using water-based electrode slurries with tailored polysaccharide binders offers an environmentally benign and cost-efficient approach to produce high performance sulfur cathodes with tremendous potential for immediate translation to industrial production.

## Methods

### Materials

The cathode was comprised of elemental sulfur (Sigma-Aldrich), carbon (CABOT black pearl 2000, Shandong Gelon LIB Co., LTD, China), Carboxymethylcellulose binder (Sigma-Aldrich) and glucose (Sigma-Aldrich). X-ray powder diffraction (XRD) spectra of aforementioned materials are shown in Supplementary Fig. 17. Carbon coated glass fibre interlayer comprised of carbon (ASAC30, Adven Industries Inc., Canada), Gum Arabic (HawkinsWatts) and glass fibre (BG03013 separator, Hollingsworth & Vose, USA). Bis (trifluoromethane) sulphonamide lithium salt and lithium nitrate were purchased from Sigma-Aldrich and directly used without any further purification. DME and DOL solvent was purchased from Sigma-Aldrich. Li_2_S was purchased from Alfa Aesar for lithium polysulfide synthesis. Battery-grade Al foil was purchased from Japan Capacitor Industrial Co. Celgard 2730 separator was purchased from Celgard Inc., USA. CNT was purchased from Nano Fibers, UK. Lithium chips (16*0.2 mm) was purchased from Shandong Gelon LIB Co., LTD, China.

### Preparation of the sulfur–carbon composite electrodes

The first step of slurry preparation was dry mixing of all components by a magnetic stirring bar (600 rpm, room temperature and dry environment) in the following order. Sulfur and conductive carbon powder were mixed for 24 h, followed by adding different kinds of binder powder (CMC and/or glucose, amount of usage listed in Table 1) to the mixture and continuing the dry mixing of all three components for another 24 h. Then, 3 mL of deionised (DI) water was added to the 1 g of well-mixed components. All components were mixed in water with a magnetic stirring bar (600 rpm, room temperature and air environment) for 12 h to make a homogenous slurry. All sulfur cathode slurries were coated by a lab-scale doctor blade on a battery-grade Al foil and dried at room temperature for 6 h, followed by 12 h drying at 80 °C under vacuum to remove all traces of solvent. Calendering processing was not applied for electrodes before both coin and pouch cells assembly. For comparison among cathodes with various binder composite, there are four cases of cathodes explained in Table 1. The sulfur loading for the coin cell cathode (1 cm × 1 cm) was from 2 mg cm^−2^ to 11 mg cm^−2^, and the sulfur loading for both single-side and double side coated cathodes (3 cm × 5 cm) was around 4 mg cm^−2^.

### Coin cells assembly and electrochemical tests

The glass fibre interlayer (0.203 mm thickness, 16 mm diameter and 15.5 µm max pore size) was coated with an aqueous slurry mixture of 80 wt. % carbon and 20 wt. % Gum Arabic (8 mL of deionised (DI) water was added to the 1 g of well-mixed components), acting as an conductive layer on sulfur cathode. To cooperate with sulfur cathode with different sulfur loading, the mass of carbon content on the aforementioned carbon coated glass fibre interlayer was 1 mg cm^−2^ for sulfur cathode with a sulfur loading of 3 mg cm^−2^, 1.5 mg cm^−2^ for sulfur cathode with a sulfur loading of 6 mg cm^−2^, and 2 mg cm^−2^ for sulfur cathode with a sulfur loading of 11 mg cm^−2^. Therefore, the total sulfur content including sulfur cathode and conductive interlayer was 56.7–62.1% (Supplementary Fig. 18). A Celgard separator (Celgard 2730, 20 µm thickness, 16 mm diameter, 1 µm pore size, and 43% porosity) was used as the separator. A schematic diagram of cell configuration is shown in Supplementary Fig. 19. The electrolyte (<0.003% water content) was prepared by dissolving 1 M Bis (trifluoromethane) sulphonamide lithium (LiTFSI) and 0.5 M lithium nitrate (LiNO_3_) in DOL and DME (1:1, v/v) in Ar-containing glovebox (<0.1 ppm H_2_O and <0.1 ppm O_2_). The electrolyte to sulfur ratio was in the range of 8.6–22 μL mg^−1^, depending on the S loading. For example, for the cathode at 3 mg cm^−2^, 15 μL of electrolyte was used to wet the cathode. To wet the carbon coated glass fibre and Celgard separator, 50 μL of electrolyte was used. For the cathode with 6 mg cm^−2^, 20 μL of electrolyte was used to wet the cathode. To wet the carbon coated glass fibre and Celgard separator, 60 μL of electrolyte was used. For the cathode with 11 mg cm^−2^, 25 μL of electrolyte was used to wet the cathode. To wet the carbon coated glass fibre and Celgard separator, 70 μL of electrolyte was used. Typically, increased amount of electrolyte was used for increased sulfur loading of the cathode. A summary of E/S ratio is shown in supplementary Table 4. E/S ratio larger than 20 μL mg^−1^ is defined as electrolyte-flooded conditions and E/S ratio lower than 5 μL mg^−1^ is defined as lean-electrolyte conditions.

For coin cell assembly, all the steps were conducted in argon glovebox and electrochemical tests were done by EC-lab (Bio-logic) under air atmosphere and room temperature. EIS measurements were conducted by potentiostatic signal with 1 mHz to 1 MHz frequency range, 6 data points pre decade of frequency, 10 mV rms alternating current (AC) voltage and 2.8 V vs E_ref_ direct current (DC) voltage.

### Pouch cell preparation

Sulfur electrodes with around 4 mg cm^−2^ were cut to be 3 cm × 5 cm (cathode and Al substrate). For double-sided electrodes, sulfur slurry was coated on the back of single-sided electrodes, yielding some sulfur loading (around 4 mg cm^−2^) on both sides. Li foil (0.1 mm thickness) was cut to the same size (3 cm × 5 cm) as sulfur cathode. The Al tab was welded on the as prepared cathode, and a Ni tab was adhered on Li anode by conductive Cu tap. After that, carbon coated glass fibre interlayer or carbon nanotube (CNT) paper interlayer was stacked on Celgard separator, followed by the cathode on the top of the interlayer. Then, a piece of Li anode was placed on another side of Celgard separator. Certain amount of electrolyte (Supplementary Table 4) was injected into the stack. Then, the package was sealed under vacuum. All cells were assembled in Ar-containing glovebox (<0.1 ppm H_2_O and <0.1 ppm O_2_). The cycling performance of coin cells configured with CNT interlayer are shown in Supplementary Fig. 20.

### Synthesis of lithium polysulfide solution

The synthesis of Li_2_S_6_ solution was followed from the method reported by Kaiming^61^. Elemental sulfur and Li_2_S powder were mixed in a solvent of DOL and DME solvent (DOL/DME = 50/50 (v/v)) at 50 °C for 36 h under stirring in an Argon glove box and with a molar ratio of 8:5 (8*Li*_2_ *S* + 5*S*_8_ → 8*Li*_2_*S*_6_). The resultant was centrifuged at 3610.7 × *g* for 10 min to abandon the particles inside, and the remaining red-brown solution is the Li_2_S_6_ solution.

### Scanning electron microscopy imaging and EDX mapping

The fresh cathode samples were mounted on Al stub with conductive carbon tap and coated with iridium for the front section and cross-section imaging. Nova 450 field emission scanning electron microscope (FESEM) and Magellan 400 FESEM were used for secondary electron imaging and energy dispersive spectroscopy mapping (EDX). EDX mapping of fresh sulfur cathode is shown in Supplementary Fig. 21. For ex situ post-mortem SEM studies, all cells were stopped at full charge before disassembling in an Argon glovebox. Cycled electrodes (cathode and anode) were washed with 1 ml of DOL/DME (1:1, v/v) and vacuum dried for 12 h before mounting on Al stubs with conductive carbon tap in Ar glovebox. The transfer vacuum module was used to transfer cycled electrodes from Ar glovebox to Merlin FESEM.

### Tensile and indentation tests

The mechanical tests were carried on these two iridescent films. The Instron 5965 with 5 kN load capacity was utilised for the tensile test. Wedge grips were applied to hold the specimens; rubber inserts were placed inside the grips to prevent tearing of films. The CMC and CMC with glucose films were cut into 5 cm long strips with a width equal to 2 cm rectangular shape with the same thickness (24 µm). The initial gage length was set at 3 cm. Tests were done under displacement control, dry conditions. Strain rates were 1 mm/min. The hardness data was obtained by Duramin A-300 Micro-Hardness tester, 0.1 N load was applied with 10 s loading time. Each sample was repeated the indentation step ten times and got the average value.

### Density tests

Gas pycnometer (Micromerities; AccuPyC II 1340) was used to measure the density of cathode components mixture and binder liquid.

### Rheology tests

Rheological tests were done using a strain-controlled ARES G2 rheometer (TA instruments, USA) using a cone and plate geometry (dia-50 mm, cone angle-2°). A constant gap of 0.045 mm and temperature of 23.00 ± 0.01 °C was maintained during the measurements. For steady-state measurements, viscosity change as a function of shear rate ranging from 0.1 to 100 s^−1^ was recorded. The amplitude sweep was done at an angular frequency of 10 rad/s, in a range from 0.1 to 100% strain amplitude, to determine the linear viscoelastic (LVE) regime. Frequency sweep was done over the range of 0.1–100 rad/s.

### Pendant drop tensiometry

The measurement of surface tension of cathode slurries was carried out using a customised pendant drop setup running OpenDrop software version 1.2. For measurement, a stable droplet of the slurry was created through a 2.7 mm outer diameter stainless steel blunt-tipped needle. The software recorded the value of surface tension every 5 seconds for a period of 250 s.

### Raman spectroscopy

Raman spectra were obtained using a Renishaw inVia Raman Spectrometer equipped with 632.8 nm HeNe laser excitation operating at 10% power with a laser spot size of 1 μm and an accumulation time of 30 s. Extended scans were performed and spectra were recorded over 180–600 cm^−1^ range. A 100 µm slit was employed.

### Fourier transform infrared spectroscopy

In order to examine the functional groups from the solid residues, Fourier Transform Infrared Spectroscopy (FTIR) spectra were recorded using an attenuated total reflectance FTIR spectrometer (PerkinElmer, USA) in the range of 400–4000 cm^−1^ at an average of 32 scans.

### UV–visible spectroscopy

The concentration of lithium sulfide (Li_2_S_6_) applied in the UV–vis test is 6 mmol/L, in DOL/ DME (1:1 v/v). 50 mg of polymer binder was soaked in 6 ml lithium polysulfide in DOL/DME electrolyte in a UV quartz container. The spectra were collected through the Thermo Scientific Evolution 220 UV–Visible Spectrophotometer during the 24-h period.

### Computational method

We carried out all density functional theory (DFT) calculations using the Vienna Ab Initio Simulation Package (VASP)^62,63^. The projector augmented wave (PAW)^64^ pseudopotentials are utilized to describe core and valence electrons, and the generalized gradient approximation based on the Perdew–Burke–Ernzerhof (GGA-PBE)^65^ function is used to describe electron exchange and correlation. Dipole correction is also considered here to avoid any spurious interactions between molecules. We select the plane-wave based kinetic energy cutoff of 450 eV, and the Γ-centered 1 × 1 × 1 Monkhorst-Pack^66^ k-point mesh for sampling the Brillouin zones of polysulfide and organic molecules without and with site adsorption. The simulation boxes of 30 Å × 30 Å × 30 Å and 10 Å × 45 Å × 45 Å are set for glucose molecules without and with adsorbed polysulfide molecules.

### NMR Spectroscopy

NMR experiments were performed on Bruker Avance 400 MHz NMR spectrometers. NMR experiments were performed with the sample held at 25 ± 0.1 °C for routine analysis. Chemical shifts for all experiments are referenced using the Unified Scale relative to 0.3% tetramethylsilane in deuteriochloform^67,68^. Samples for NMR spectroscopy were prepared by dissolving the analyte in deuterated solvent, as specified, and placing the solution into a 5 mm NMR tube. The data were processed using Bruker TopSpin v3.6.2 software.

## Supplementary information

Supplementary Information

## Data Availability

All data needed to evaluate the conclusions in the paper are present in the paper and/or the Supplementary Information/Source Data file. Additional data related to this paper may be requested from the authors. Source data are provided with this paper.

## Source data

Source data
