# Supplementary material for: A saccharide-based binder for efficient polysulfide regulations in Li-S batteries
Source: Nat Commun. 2021 Sep 10;12:5375. doi: 10.1038/s41467-021-25612-5 (PMC8433142; doi:10.1038/s41467-021-25612-5)
Supplement: Supplementary file 1 — Supplementary Information [file 41467_2021_25612_MOESM1_ESM.pdf]

Supplementary Information

A saccharide-based binder for efficient polysulfide regulations in Li-S batteries

Yingyi Huang[1], Mahdokht Shaibani[1*], Tanesh D. Gamot[1], Mingchao Wang[2], Petar Jovanović[1], M. C. Dilusha Cooray[1], Meysam Sharifzadeh Mirshekarloo[1], Roger Mulder[4], Nikhil V. Medhekar[2], Matthew R. Hill[3,4*], Mainak Majumder[1*]

[1] Nanoscale Science and Engineering Laboratory (NSEL), Department of Mechanical and Aerospace Engineering, Monash University, Clayton, VIC 3168, Australia

[2] Department of Materials Science and Engineering, Monash University, Clayton, VIC 3168, Australia

[3] Department of Chemical Engineering, Monash University, Clayton, VIC 3168, Australia

[4] CSIRO, Clayton, VIC 3168, Australia

[*]Email: m.e.shaibani@monash.edu, matthew.hill@csiro.au, mainak.majumder@monash.edu

Supplementary Figures

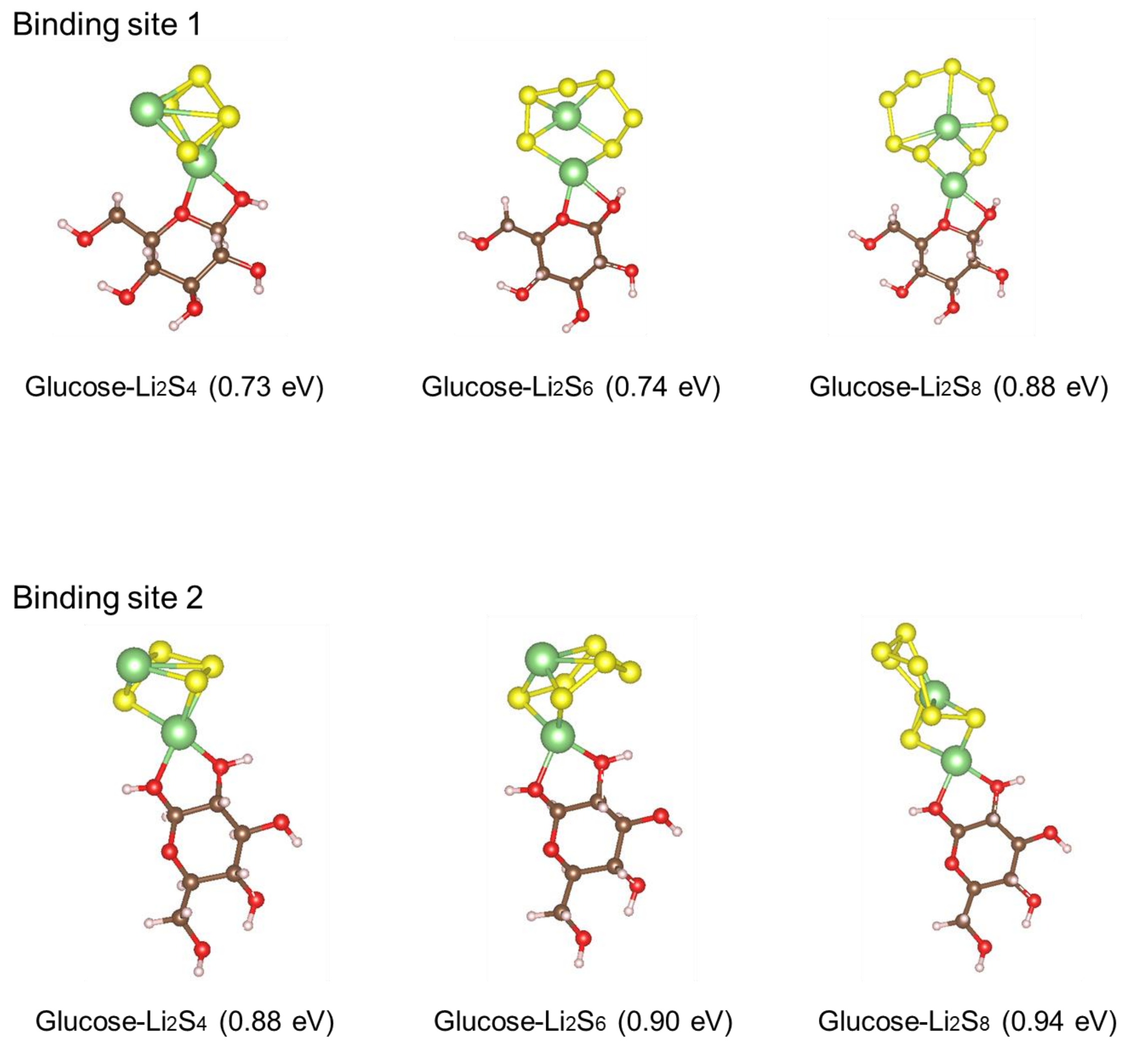

Supplementary Figure 1. Adsorption conformations and binding energies for Li_2S_4, Li_2S_6, and Li_2S_8 on glucose (other two possible binding sites).

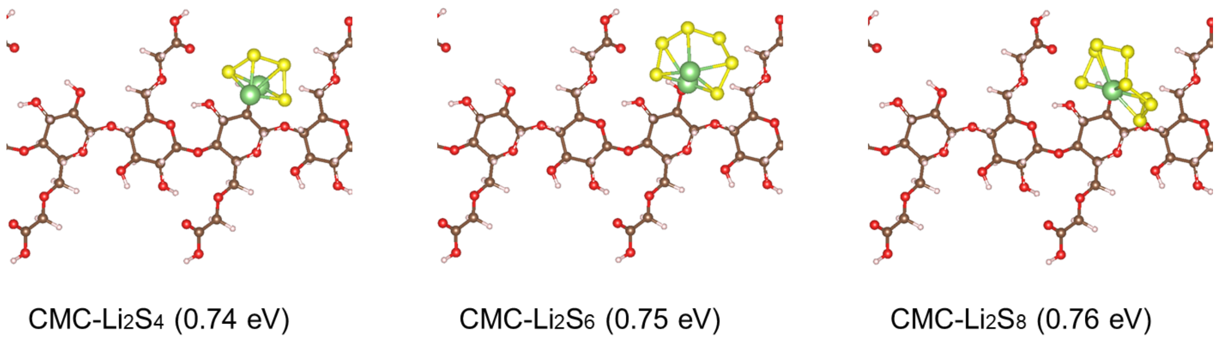

Supplementary Figure 2. Adsorption conformations and binding energies for Li_2S_4, Li_2S_6, and Li_2S_8 on CMC. The binding energies with CMC (0.74-0.76 eV) are relatively lower than the binding energies with glucose (0.90-0.95 eV), but the difference is not obvious when compared to the experimental absorption test. We attribute this inconsistency with experiments to several plausible reasons: (a) In experiments, kinetics of LiPS diffusion is likely to be very different for glucose and CMC. However, this aspect is ignored in our calculations, which simply estimate the strength of binding interactions. (b) The calculations for binding energy assume an ideal geometry at the atomic scale, assumptions that may not hold when comparing with experiments.

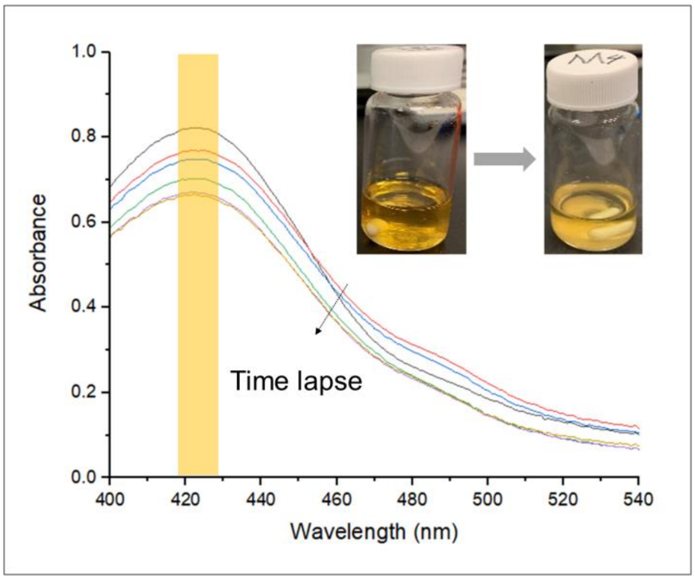

Supplementary Figure 3. UV-Vis spectrum of Li_2S_6 with CMC in DOL/DME electrolyte solution after certain time and evolution of CMC with Li_2S_6 in DOL/DME electrolyte solution.

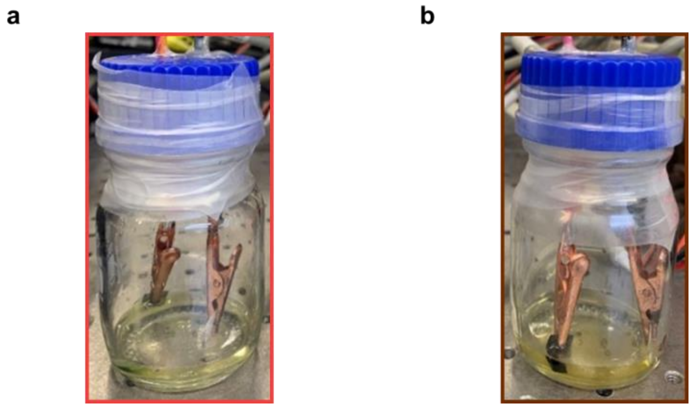

Supplementary Figure 4. Glass transparent with lithium anode and sulfur cathode immersed in electrolyte after cycling. **a** CMC/G cathode and **b** CMC cathode.

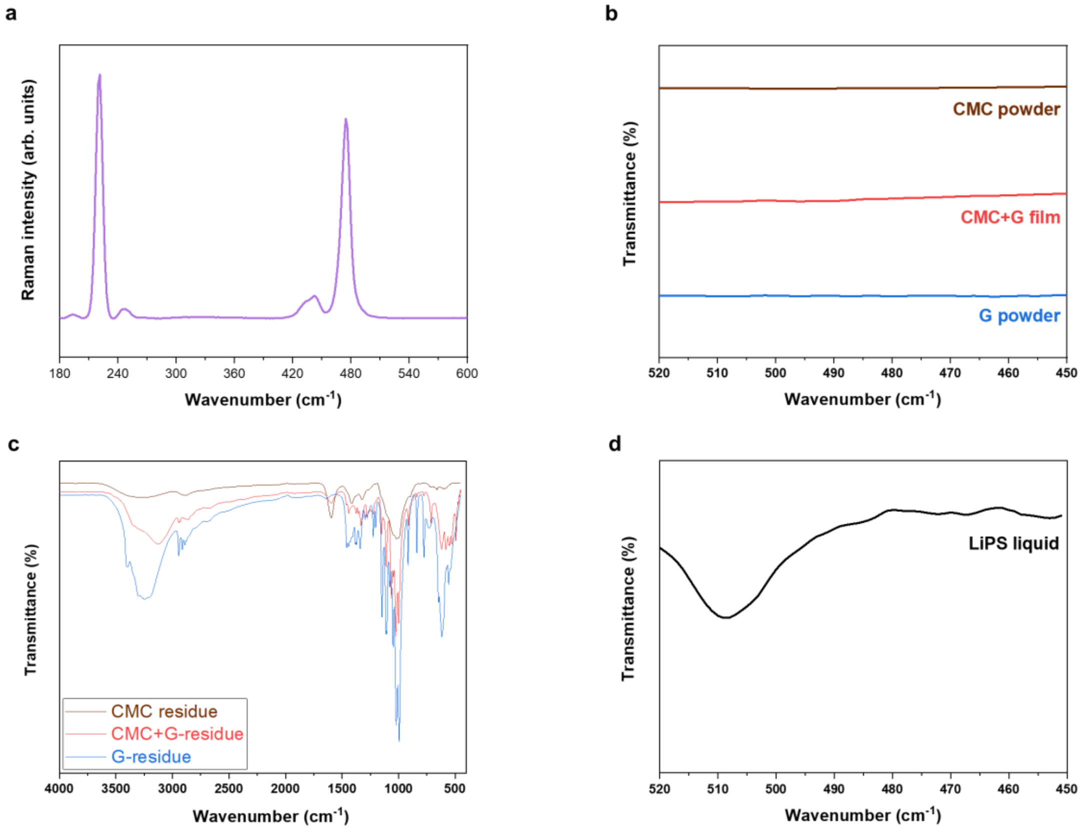

Supplementary Figure 5. Raman and FTIR test. **a** Raman spectra for liquid LiPS reactant. **b** FTIR spectra for binder ingredients. **c** Full FTIR spectra for residue samples. **d** FTIR spectrum for liquid LiPS.

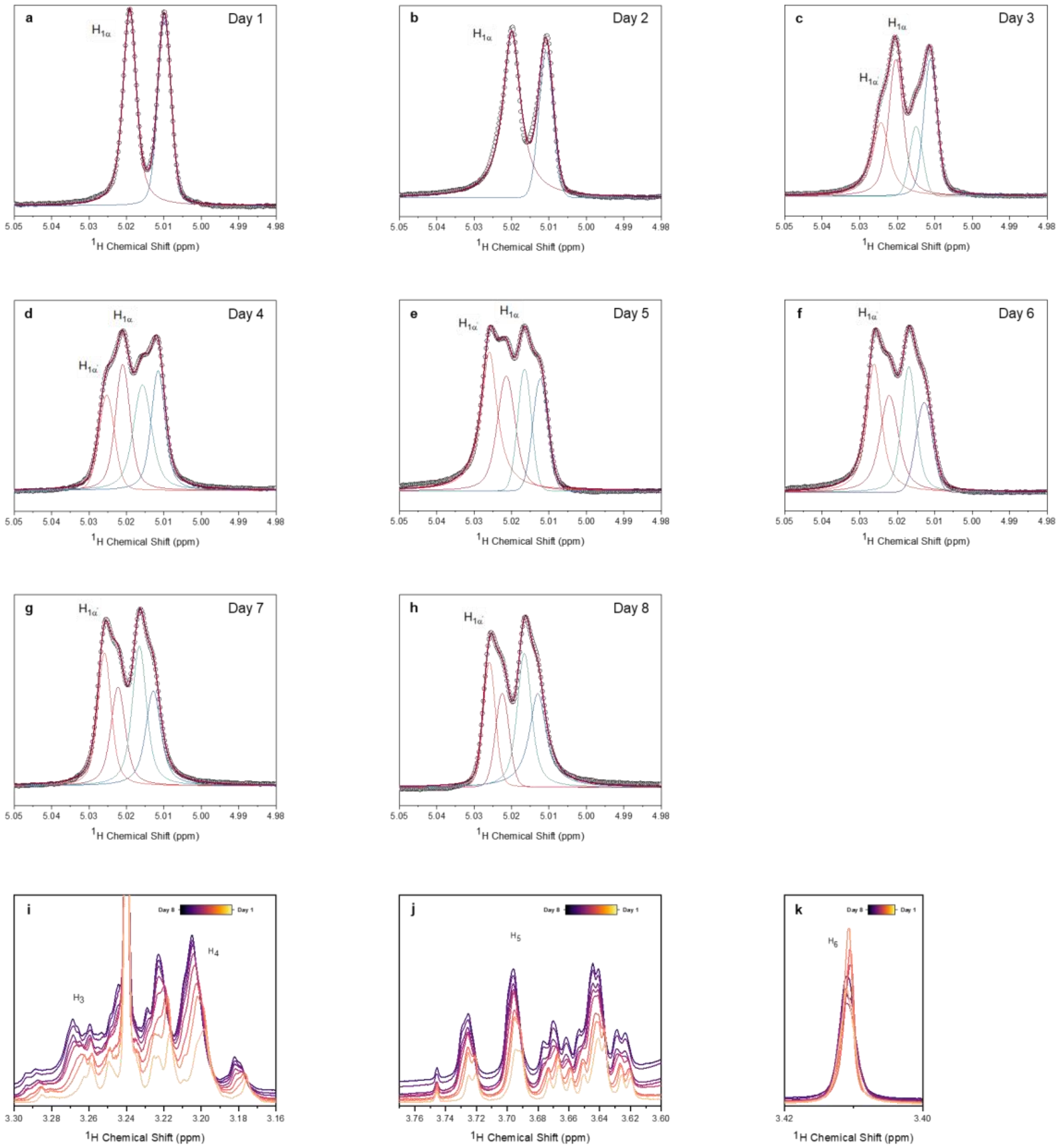

Supplementary Figure 6. In depth ^1H NMR analysis of the glucose- Li_2S_6 interaction within a simulated battery environment.

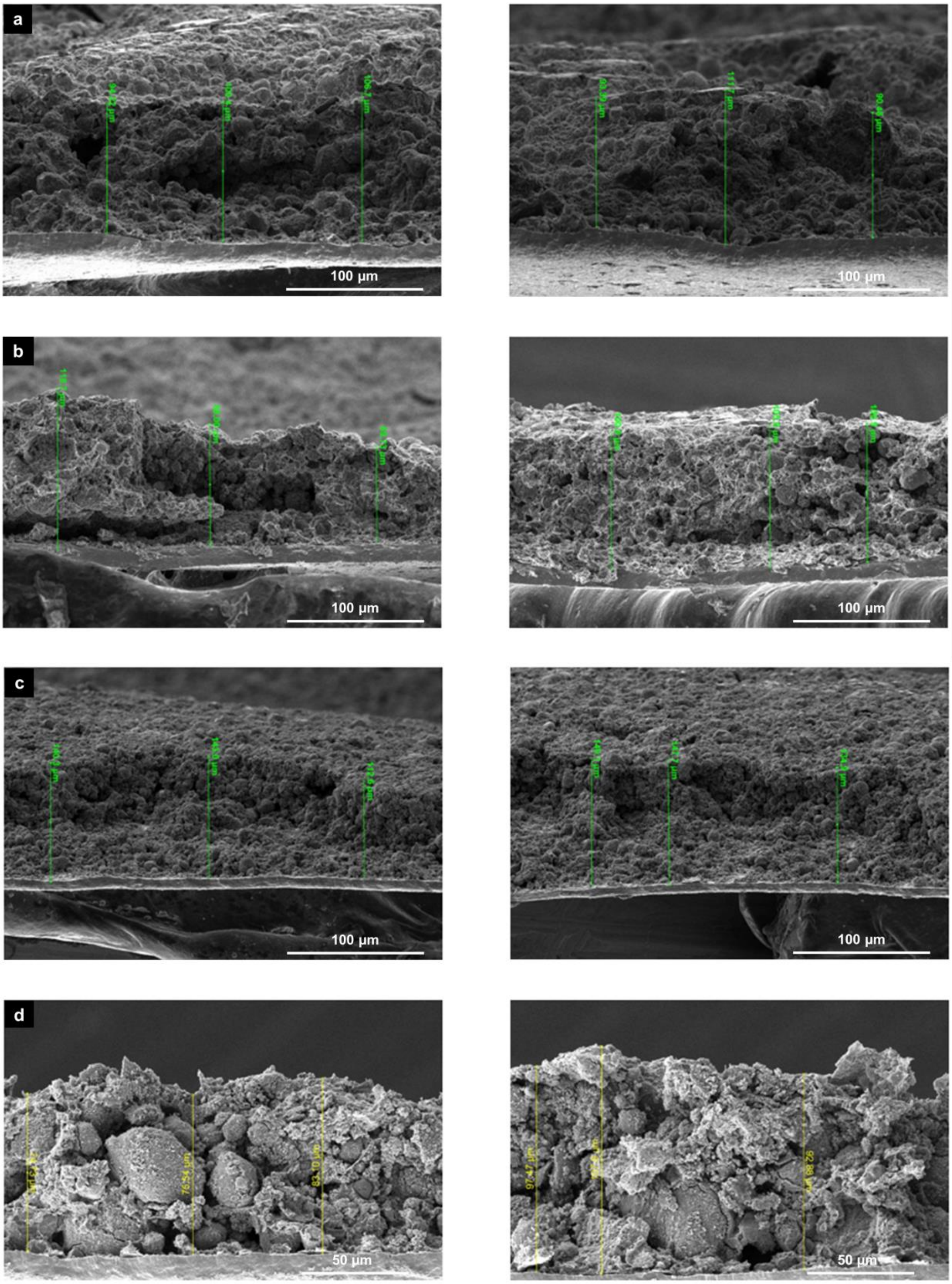

Supplementary Figure 7. Cross-section of cathodes with **a** Pure CMC, **b** 0.67CMC + 0.33G, **c** Pure glucose and **d** 0.5CMC + 0.5G and as binder.

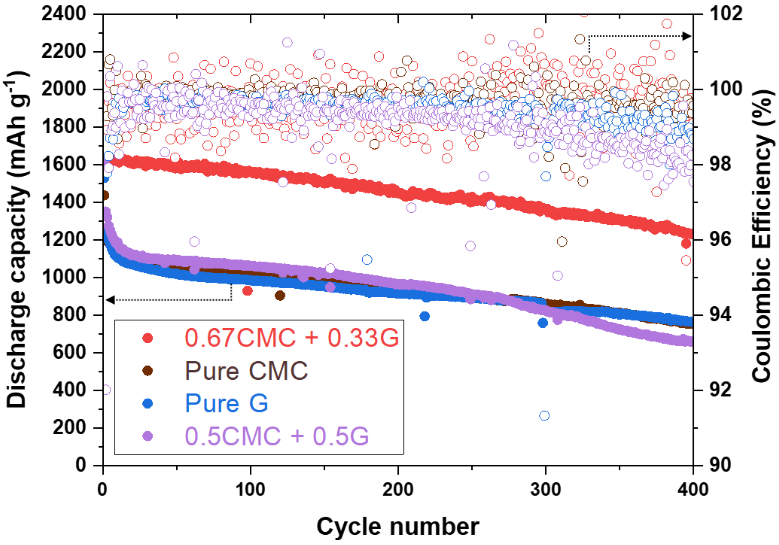

Supplementary Figure 8. Cycle performance of electrodes with different binders.

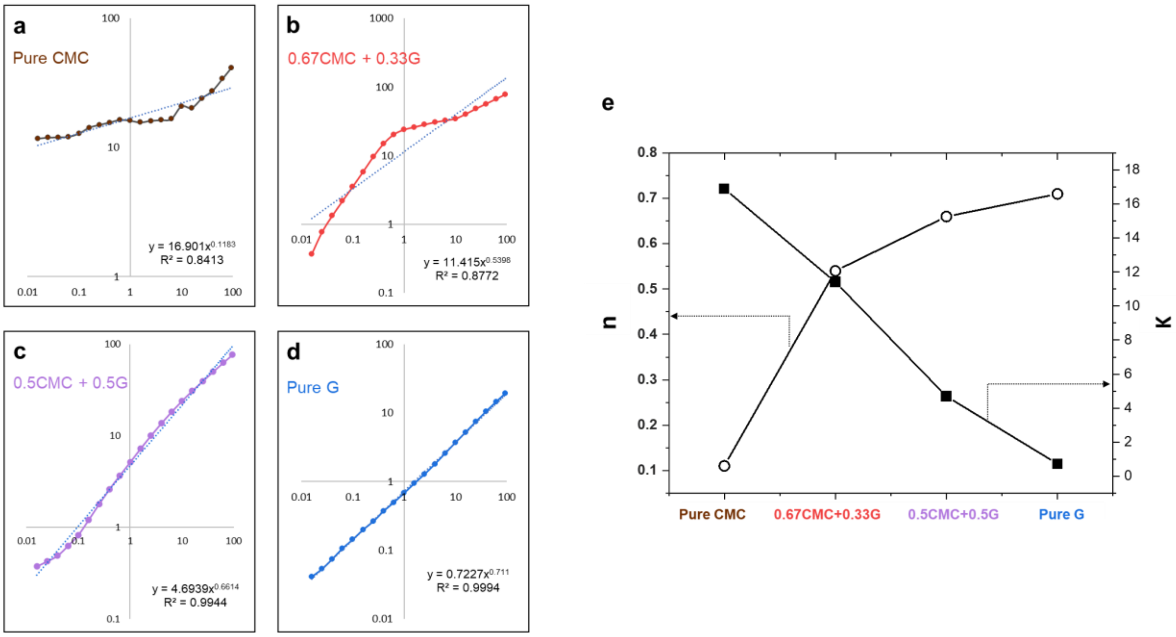

Supplementary Figure 9. Power law calculation of **a** Pure CMC, **b** 0.67CMC + 0.33G, **c** 0.5CMC + 0.5G and **d** Pure glucose cathode slurries. **e** Flow behaviour index n and flow consistency index K (represents limit of viscosity of fluid at an infinite shear stress) of the cathode slurries determined using the power law.

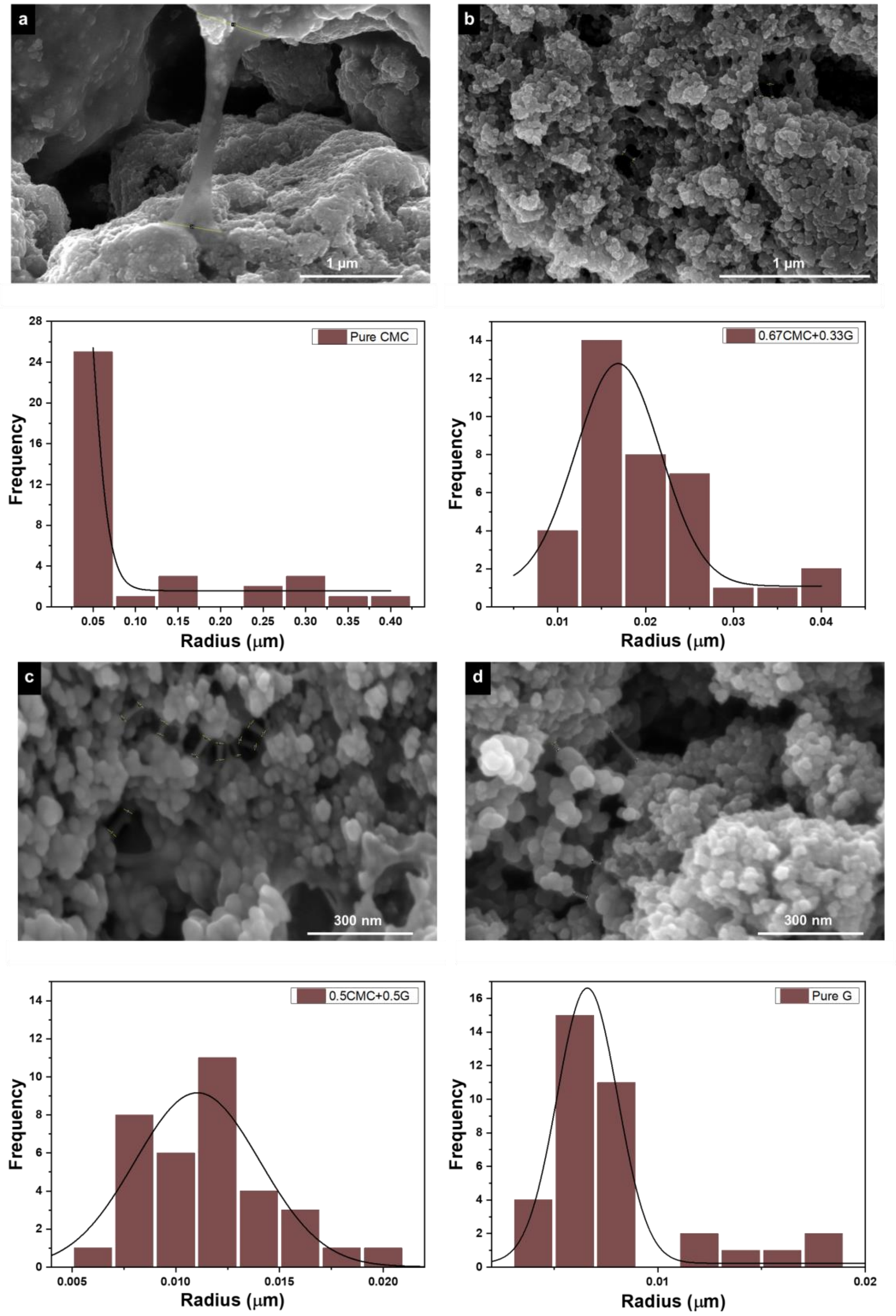

Supplementary Figure 10. SEM of sulfur cathode for binder filaments initial radius calculation. Histogram of binder filaments initial radius with the Gaussian distribution fitting. **a** Pure CMC, **b** 0.67CMC + 0.33G, **c** 0.5CMC + 0.5G and **d** Pure glucose as binder.

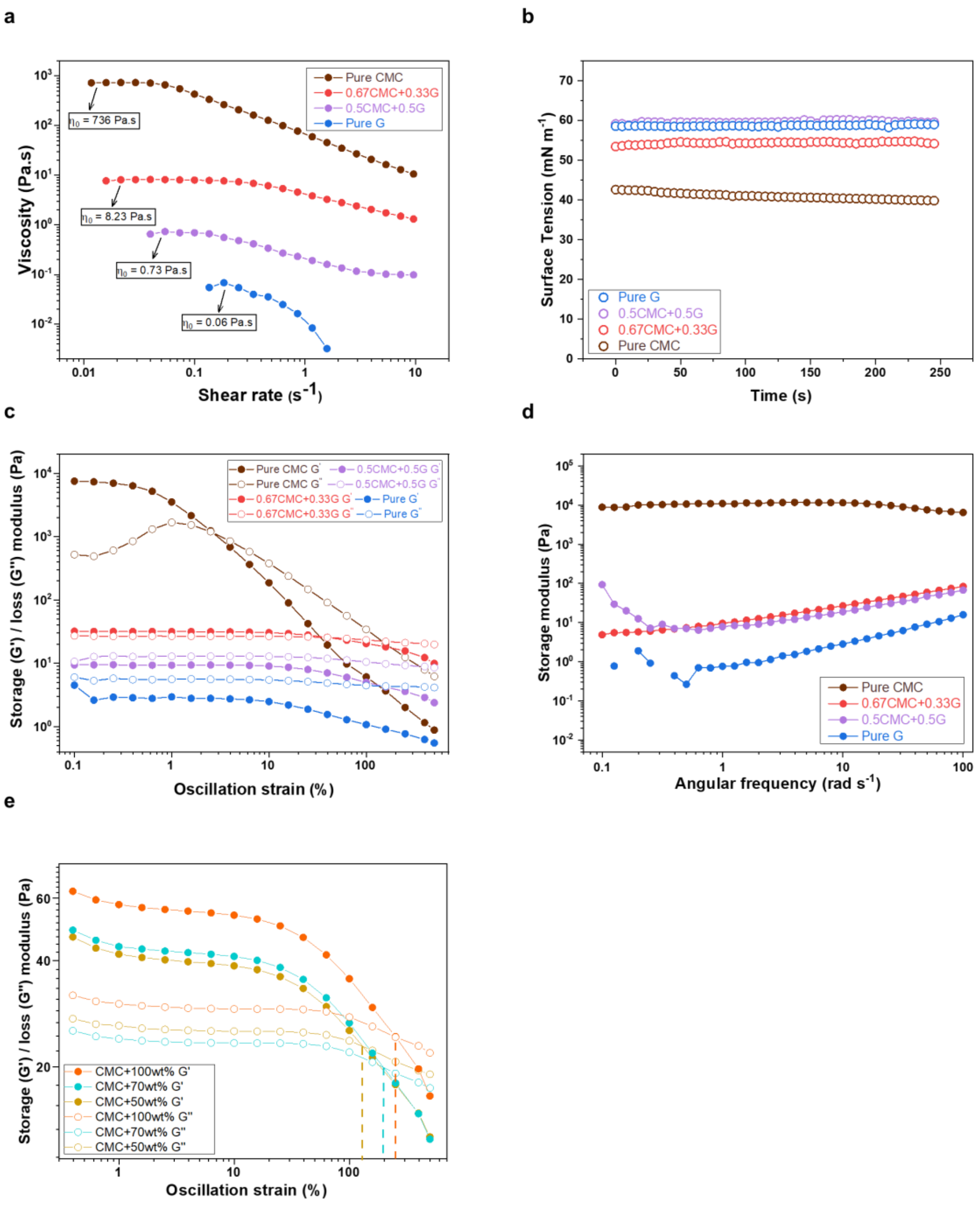

Supplementary Figure 11. a Zero-shear viscosity and **b** Surface tension of four binders. Viscoelastic properties of the slurries **c** Amplitude sweep measurements and **d** Frequency sweep measurements of four different sulfur cathode slurries. **e** Amplitude sweep measurements of CMC and CMCG binder itself with different solid content in water.

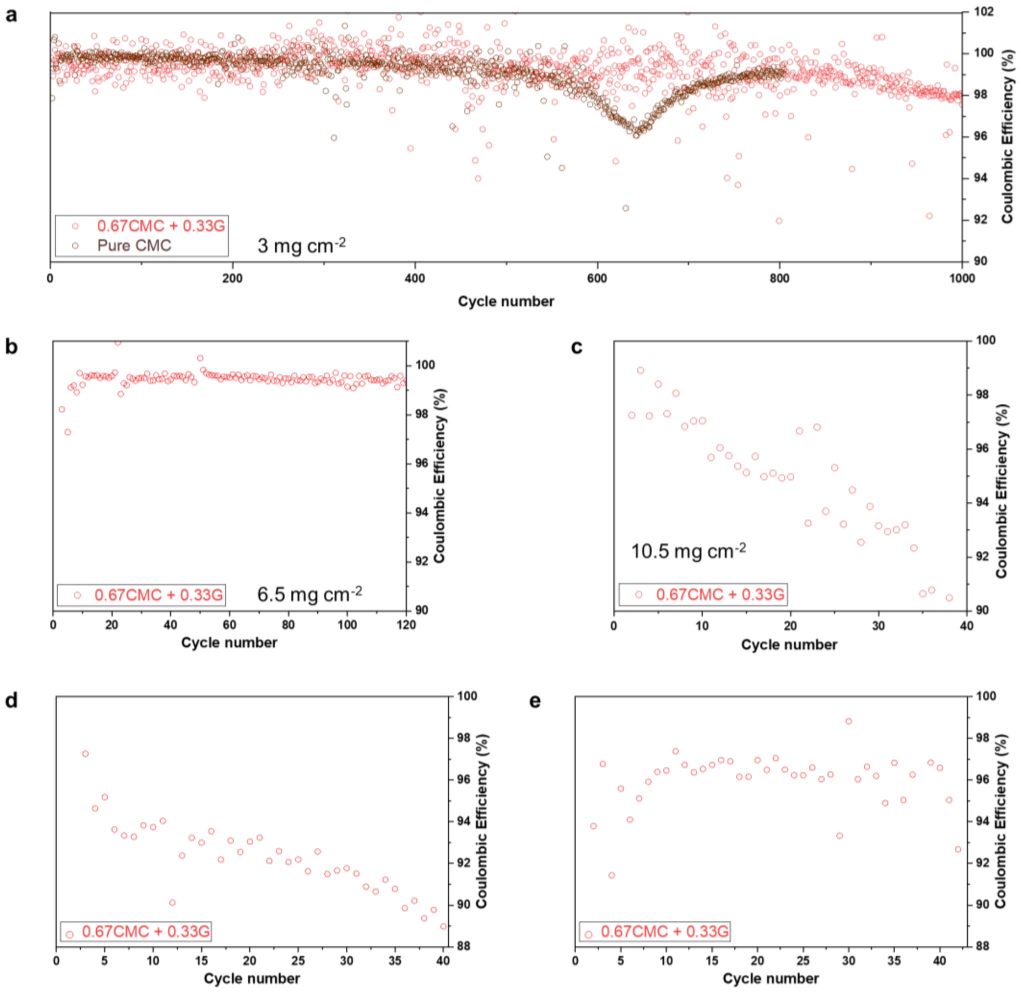

Supplementary Figure 12. Coulombic efficiency of CMC cathode and CMC/G cathode. Coin cells configured with **a** 3 mg cm^{-2} sulfur loading cathode, **b** 6.5 mg cm^{-2} sulfur loading cathode and **c** 10.5 mg cm^{-2} sulfur loading cathode. Configuration of the pouch cell with **d** single-sided cathodes **e** double-sided cathodes with leaner electrolyte condition (E/S= 6 μL mg^{-1}), optimised for specific energy. The fluctuations in the CE of the CMC/G cathode after a few hundreds of cycles can be attributed to three plausible reasons: (1) the phase transitions between solid elemental sulfur, liquid higher order of lithium polysulfide and solid lower order of lithium polysulfide are not fully achieved in some certain discharges or charges due to the gradual consumption of the electrolyte over long term cycling. During the battery system re-equilibration, the coulombic efficiency is sometimes larger than 100 % until the completion of the capacity recovery cycles, then returned to around 100 %. (2) $LiNO_3$ as an effective shuttle suppressor was used as an electrolyte additive in our batteries. Its degradation products contribute to the formation of a suitable surface coating that protects Li from further reaction with LiPS and lead to high coulombic efficiencies. Nevertheless, lithium nitrate is continuously consumed during cycling which

reflects as a fluctuation in battery efficiency fluctuation at a later stage. (3) During long time cycle life (9 month in this study), localized lithium metal corrosion and passivation is inevitable reflecting in fluctuation of the coulombic efficiency.

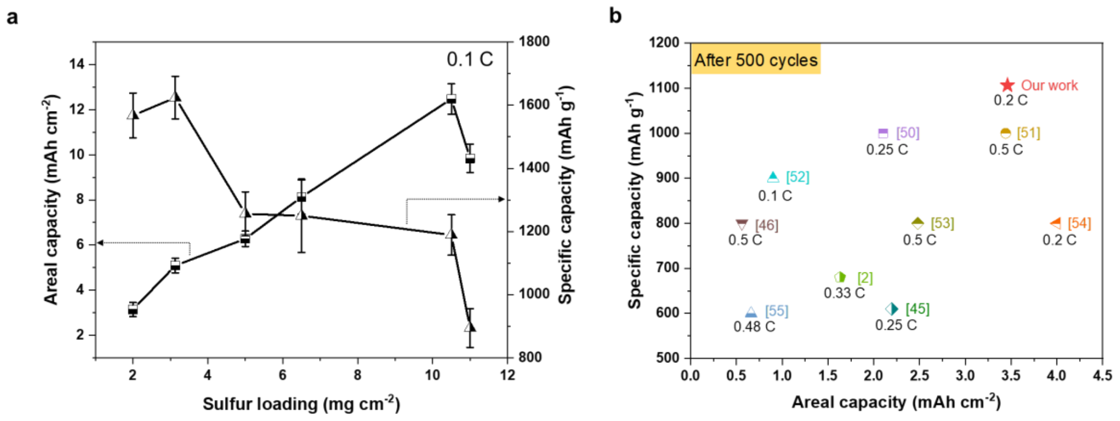

Supplementary Figure 13. a Areal and specific capacity as a function of sulfur loading of cathode in coin cells. The error bars (standard deviation) of each data point were calculated based on the cycle performance of 3 coin cells with similar sulfur loadings. **b** Comparative analysis of the areal capacity and total gravimetric capacity of the cathodes in coin cell level after 500 cycles in noteworthy literatures.

a

b

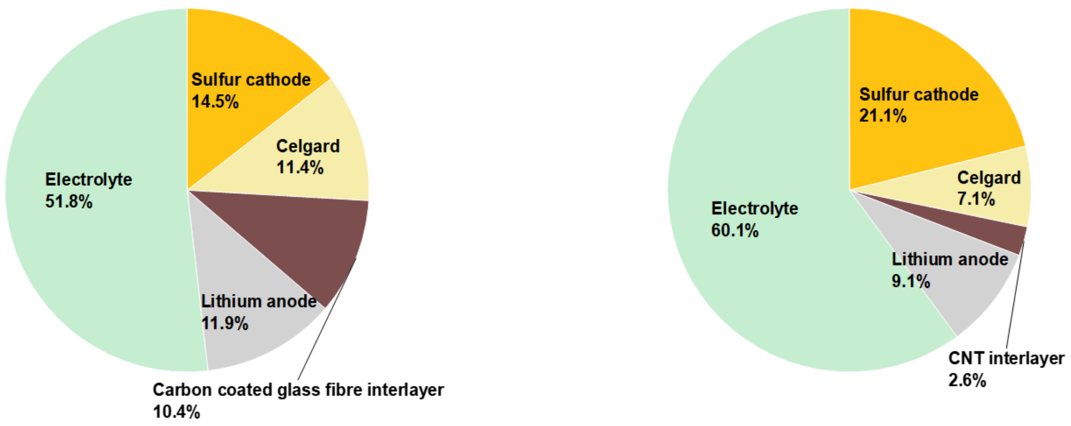

c

	Pouch cell configured with carbon coated glass fibre interlayer	Configuration optimized pouch cell using CNT paper interlayer
Total pouch weight	2894 mg	4260 mg
Dimension	3 cm (width)* 5 cm (length)* 3 single-sided layers	3 cm (width)* 5 cm (length)* 4 double-sided layers
Sulfur loading	135 mg	448 mg
E/S ratio	11 μL mg^{-1}	6 μL mg^{-1}
Capacity	194 mAh	408 mAh
Specific capacity	1200 mAh g^{-1}	910 mAh g^{-1}
Specific energy	116 Wh kg^{-1}	206 Wh kg^{-1}

Supplementary Figure 14. Proportion of each component of **a** Pouch cell configured with single-sided cathode and carbon coated glass fibre interlayer, and **b** Optimized pouch cell with double-sided cathodes and CNT paper as the interlayer. **c** Detailed information of these two pouch cells.

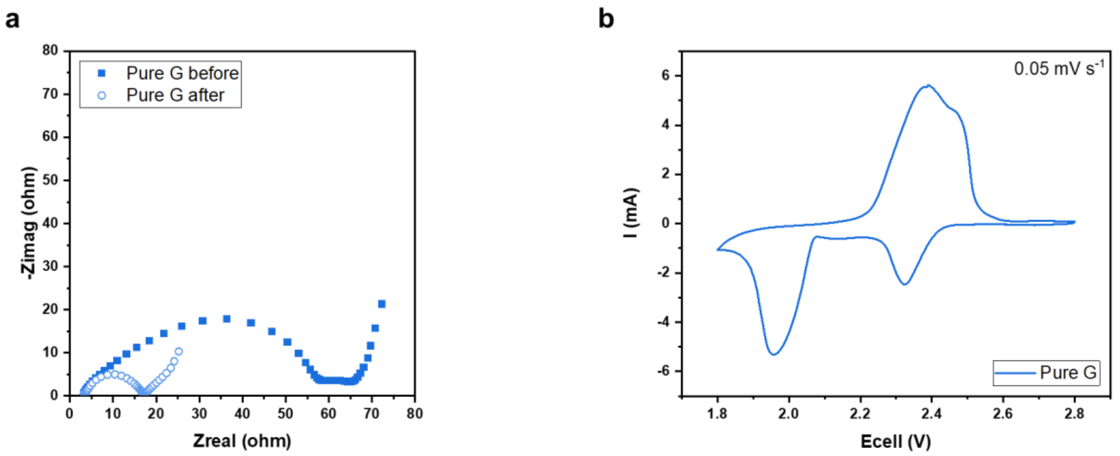

Supplementary Figure 15. Electrochemical characterisation on pure glucose cathodes. a Nyquist plots; **b** Cyclic voltammogram profiles.

a

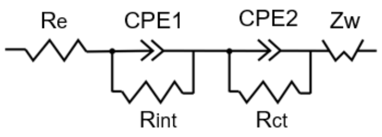

b

	R_e (ohm cm^2)	R_{int} (ohm cm^2)	CPE1 (F $cm^{-2}s^{a-1}$)	R_{ct} (ohm cm^2)	CPE2 (F $cm^{-2}s^{a-1}$)
0.67CMC + 0.33G	3.319±1.000	0.320±1.347e^{-6}	7.646e^{-3} ±1.021e^{-1}	14.290±1.00	4.437e^{-6} ±2.673e^{-6}
Pure CMC	3.431±0.478	0.541±0.660	4.074e^{-6} ±2.005e^{-3}	15.29±1.676	5.468e^{-6} ±5.581e^{-6}

Supplementary Figure 16. a Equivalent circuit. **b** Impedance parameters extracted by fitting of Nyquist plots with the equivalent circuit elements.

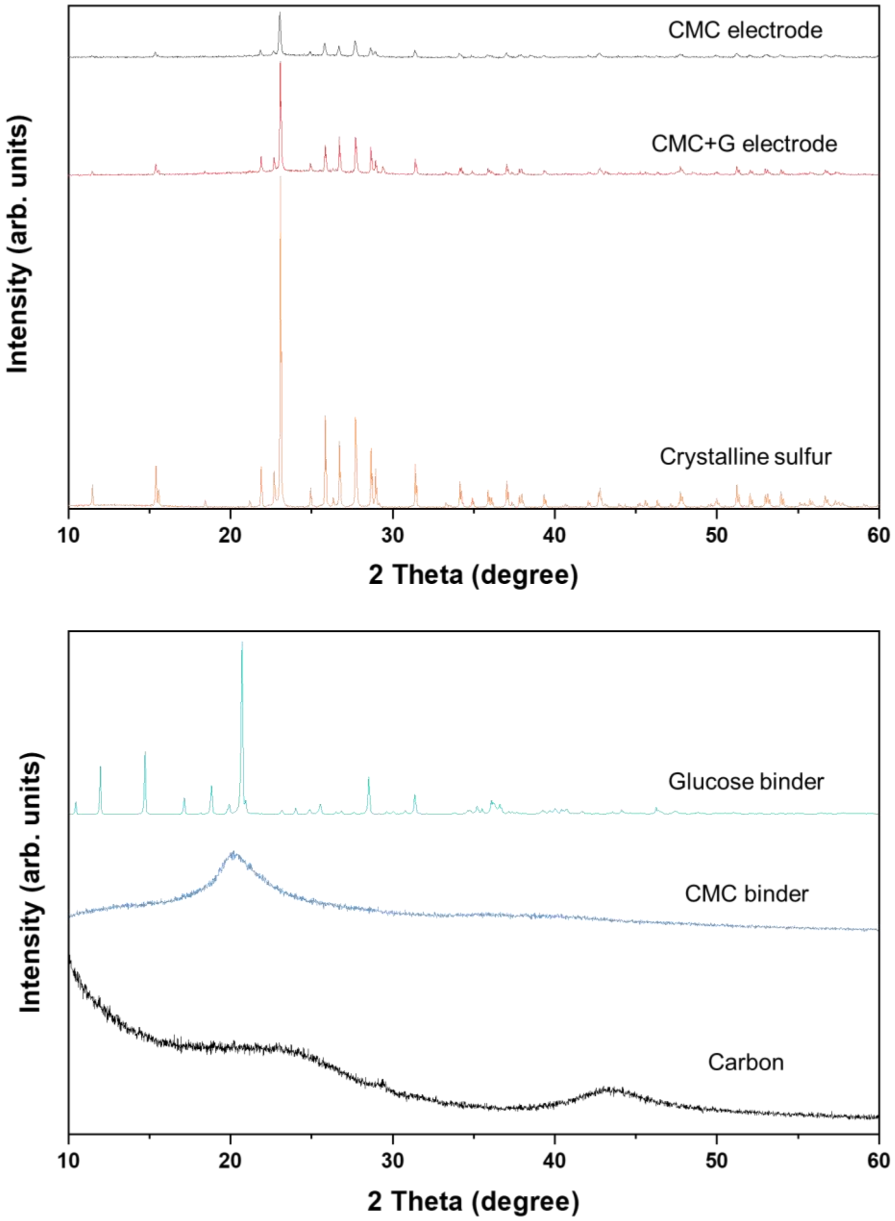

Supplementary Figure 17. XRD of electrode and associated components.

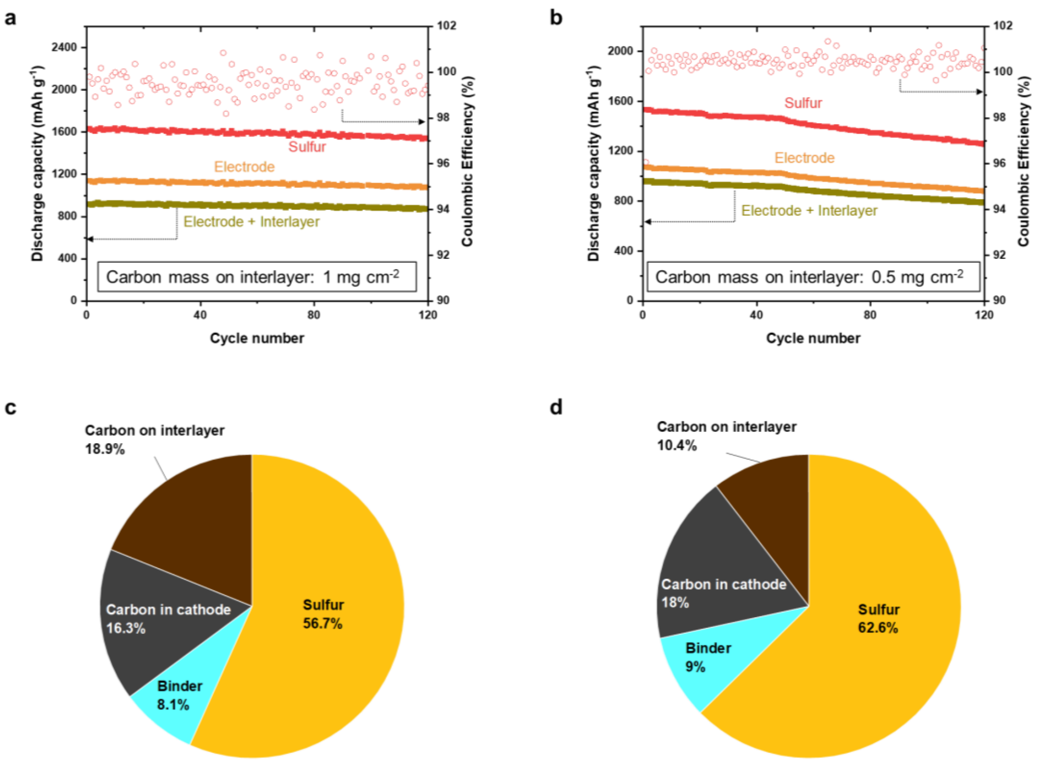

Supplementary Figure 18. Discharge capacities (0.2 C) of coin cell (3 mg cm^{-2} sulfur loading cathode) configured with **a** 1 mg cm^{-2} and **b** 0.5 mg cm^{-2} carbon coated glass fibre interlayer, based on the mass of sulfur (red lines), total mass of the electrode (light-orange line), and total mass of the electrode and additional interlayers (dark-yellow line) on the cathode side of the cell. Proportion of each component in cathodic system configured with **c** 1 mg cm^{-2} (total sulfur content including sulfur cathode and conductive interlayer was 56.7% - 62.1% based on different sulfur loading cathodes) and **d** 0.5 mg cm^{-2} carbon coated glass fibre interlayer (total sulfur content including sulfur cathode and conductive interlayer was 62.6% - 67.9% based on different sulfur loading cathodes)

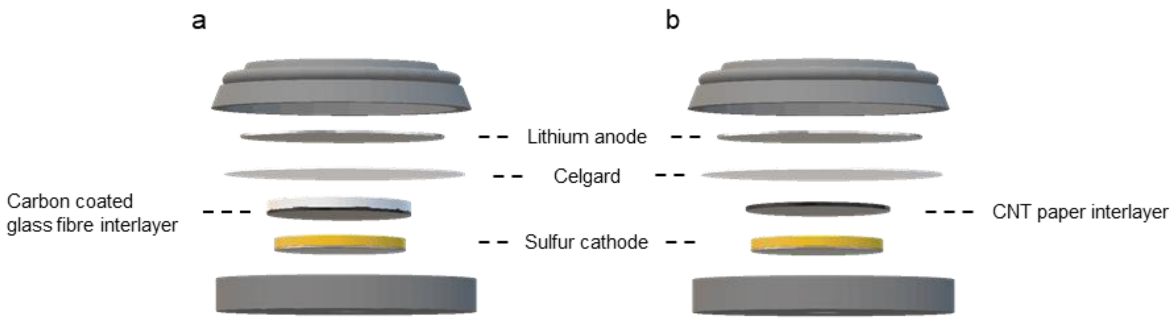

Supplementary Figure 19. Schematic presentation of **a** cells configured with carbon coated glass fibre, **b** cell configured with CNT paper interlayer.

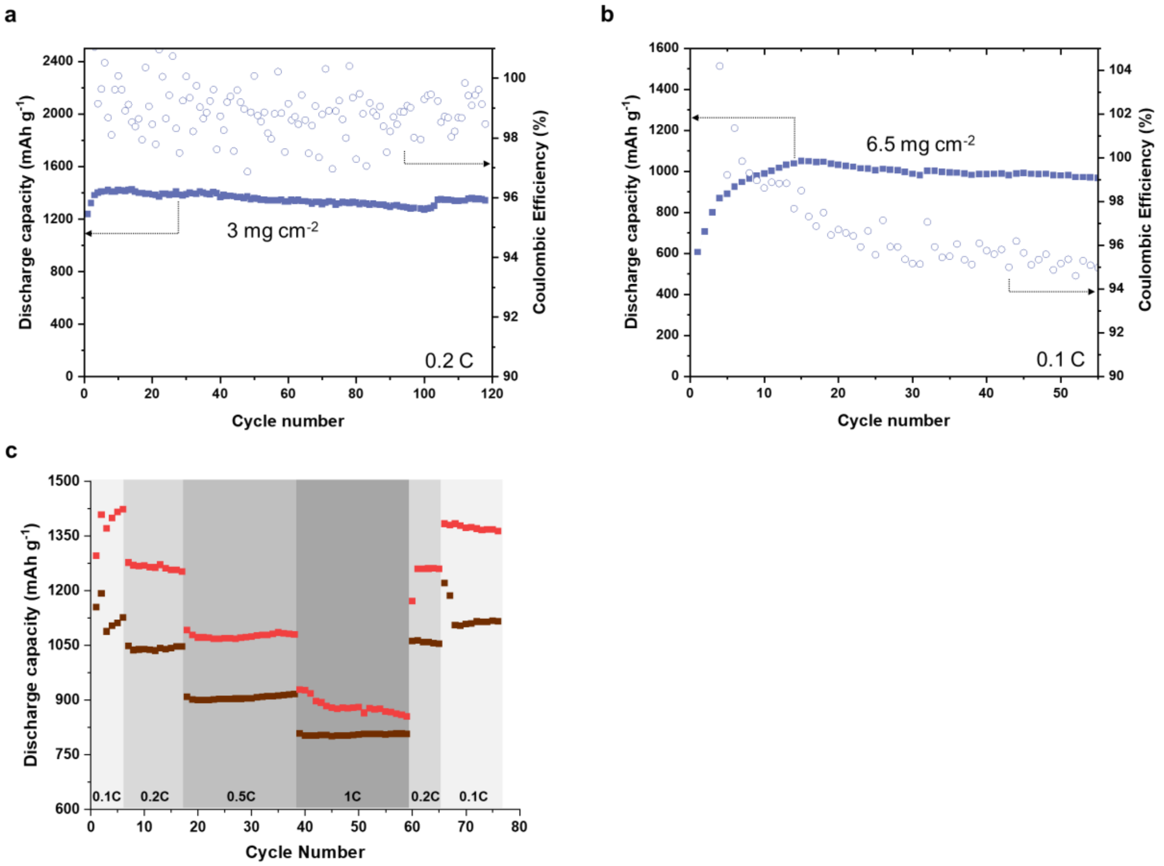

Supplementary Figure 20. Applications of CNT interlayer. Identical cells were made while replacing the carbon coated glass fibre interlayer which unduly absorbs a lot of electrolyte with an ultralight CNT (carbon nanotube) paper interlayer (0.5 mg cm-2), which advantageously acts as an upper current collector that allows for lean electrolyte conditions. In the newly made cells, the electrolyte to sulfur ratio was reduced to around 7.7-18 μL mg-1 at the coin cell level. Cycling performance of the CMC/G cathode

with the ultralight CNT interlayer for coin cells with **a** 3 mg cm^{-2} sulfur loading **b** 6.5 mg cm^{-2} sulfur loading. **c** Rate capability data among two compared samples (3 mg cm^{-2} sulfur loading for cathode) configured with CNT interlayer, red lines indicate the performance of CMC/G cathode, and the brown lines indicate the performance of CMC cathode.

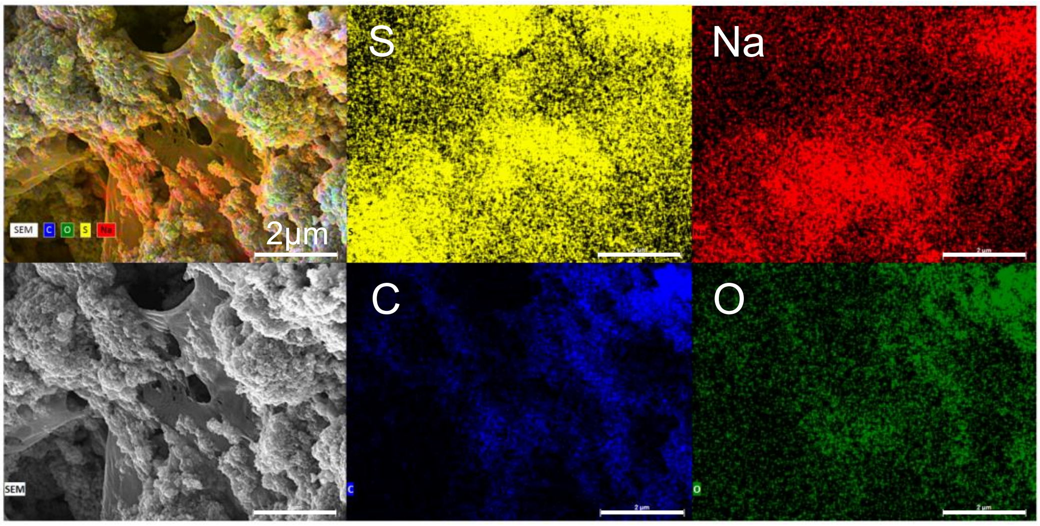

Supplementary Figure 21. EDX mapping of sulfur cathode with 0.67CMC + 0.33G as binder.

Supplementary Tables

Supplementary Table 1. Mean radius and standard deviation of distribution curves.

Slurry	Mean radius (μm)	Standard deviation
Pure CMC	0.0433	0.3024
0.67CMC+0.33G	0.0169	0.1414
0.5CMC+0.5G	0.0110	0.0691
Pure G	0.0066	0.0599

Supplementary Table 2. Density of four binders.

Trials	Pure CMC		0.67CMC+0.33G		0.5CMC+0.5G		Pure G	
	Density ($g\ cm^{-3}$)	Error	Density ($g\ cm^{-3}$)	Error	Density ($g\ cm^{-3}$)	Error	Density ($g\ cm^{-3}$)	Error
1	1.0254	0.0046	1.0241	0.0020	1.0215	0.0032	1.0223	0.0023
2	1.0260	0.0040	1.0248	0.0013	1.0223	0.0024	1.0230	0.0016
3	1.0271	0.0029	1.0254	0.0007	1.0235	0.0012	1.0238	0.0008
4	1.0307	0.0008	1.0261	0.0000	1.0243	0.0003	1.0240	0.0006
5	1.0306	0.0007	1.0262	0.0001	1.0240	0.0007	1.0244	0.0002
6	1.0311	0.0012	1.0266	0.0005	1.0261	0.0014	1.0255	0.0009
7	1.0301	0.0001	1.0266	0.0005	1.0258	0.0011	1.0252	0.0006
8	1.0328	0.0028	1.0253	0.0008	1.0263	0.0016	1.0258	0.0012
9	1.0337	0.0037	1.0280	0.0019	1.0263	0.0017	1.0257	0.0011
10	1.0322	0.0022	1.0279	0.0018	1.0266	0.0020	1.0263	0.0017
Average	1.02997	0.0023	1.0261	0.0010	1.0247	0.0016	1.0246	0.0011

Supplementary Table 3. Cycle performance comparison.

Cycle number	Areal capacity ($mAh\ cm^{-2}$)		Specific capacity ($mAh\ g^{-1}$)	C-rate	References
	1st cycle	500th cycle			
500	2.88	1.63	680	0.33	2
500	3.42	2.20	610	0.25	45
500	0.70	0.56	800	0.50	46
500	2.31	2.10	1000	0.25	50
1200	4.00	3.44	1000	0.50	51
600	1.20	0.90	900	0.10	52
500	3.41	2.48	800	0.50	53
1000	4.50	4.00	800	0.20	54
1000	0.99	0.66	600	0.48	55
1000	5.10	3.46	1106	0.20	Our work

Supplementary Table 4. Summary of E/S ratio (μL mg^{-1}) based on different interlayer configurations, sulfur loadings and cell types.

	Coin cell sulfur loading			Pouch cell
	3 (mg cm^{-2})	6 (mg cm^{-2})	11 (mg cm^{-2})	~ 4 (mg cm^{-2})
Carbon coated glass fibre interlayer	22	13	8.6	11
CNT paper interlayer	18	10	7.7	5

Supplementary Notes

Supplementary Note 1.

Equation of Ohnesorge number:

$$Oh \equiv t_v/t_c = \eta_0/\sqrt{\rho R \gamma} \quad \text{(Equation S1)}$$

η_0 : the zero-shear viscosity (Pa.s)

γ : the surface tension (mN m^{-1})

ρ : the density of the liquid (kg m^{-3})

R : the initial radius of the binder filaments (μm)

Supplementary Note 2.

Equation of binding energy simulation:

$$E_b = E_p + E_{LiPS} - E_{P-LiPS} \quad \text{(Equation S2)}$$

E_{b} : binding energy

E_{p} : isolated polymer

E_{LiPS} : isolated LiPS species

$E_{\mathrm{P-LiPS}}$: the total energy for the adsorption system

With this definition, a positive binding energy indicates a thermodynamically favourable binding.

Supplementary Note 3.

Randles-Sevick equation for lithium-ion diffusion coefficient test:

$$I_{\mathrm{p}} = 2.69 \times 10^5 n^{1.5} A D_{\mathrm{Li^+}}^{0.5} C_{\mathrm{Li}} v^{0.5} \quad \text{(Equation S3)}$$

I_{p} (A) is the peak current, n indicates the number of electrons in the redox reaction (n = 2 in Li–S batteries), A (cm^2) represents the electrode area (1 cm^2 in this work), $D_{\mathrm{Li^+}}$(cm^2 s^{-1}) is the lithium ion diffusion coefficient, C_{Li} (mol mL^{-1}) indicates the lithium-ion concentration in the electrolyte, and v (V s^{-1}) is the scanning rate.

Supplementary Note 4.

The specific energy of the pouch cells was evaluated based on the equation:

$$E_g = \frac{VC}{\sum m_i} \quad \text{(Equation S4)}$$

E_g : specific energy (Wh kg^{-1})

V : output voltage (V)

C : output capacity (mAh)

$\sum m_i$: total weight taken into consideration (including cathode, anode, conductive interlayer, separator and electrolyte)

Supplementary Note 5.

Power law:

$$\tau = K\dot{\gamma}^n \quad \text{(Equation S5)}$$

K: flow consistency index

$\dot{\gamma}$: shear rate (s^{-1})

n: flow behaviour index

τ: shear stress (Pa)
